# Comparative Effects of Quinoa, Buckwheat, Bulgur, and Rice on Cardiometabolic Health in Adults with Overweight or Obesity: A Randomized Controlled Study

**DOI:** 10.3390/metabo16090628

**Published:** 2026-08-29

**Authors:** Yesim Oztekin, Buket Gonen-Colak, Selin Tekin, Incilay Lay, Tomris Erbas, Selcuk Dagdelen, Zehra Buyuktuncer

**Affiliations:** 1 Department of Nutrition and Dietetics, Faculty of Health Sciences, Hacettepe University, Ankara 06100, Türkiye; yesimoztekin@hacettepe.edu.tr (Y.O.); bgonen@bandirma.edu.tr (B.G.-C.); 2 Department of Nutrition and Dietetics, Faculty of Health Sciences, Bandırma Onyedi Eylül University, Balıkesir 10200, Türkiye; 3 Department of Endocrinology and Metabolism, Faculty of Medicine, Hacettepe University, Ankara 06100, Türkiye; selin.tekin@hacettepe.edu.tr (S.T.); erbast@hacettepe.edu.tr (T.E.); dagdelen@hacettepe.edu.tr (S.D.); 4 Department of Medical Biochemistry, Faculty of Medicine, Hacettepe University, Ankara 06100, Türkiye; isinici@hacettepe.edu.tr

**Keywords:** pseudocereals, cereals, quinoa, buckwheat, obesity, cardiometabolic health

## Abstract

**Highlights:**

**What are the main findings?**

- The cardiometabolic effects of quinoa and buckwheat are comparable to those of rice and bulgur by significantly reducing blood pressure and body fat mass in individuals with overweight and obesity. However, the effects differ by sex, with a more pronounced reduction in blood pressure observed among women.
- The reproducible findings of this 4-week intervention indicated that none of the rice, bulgur, quinoa, and buckwheat is superior for weight management, but the results support the potential overall cardiometabolic benefits of quinoa and buckwheat consumption.

**What are the implications of the main findings?**

- Quinoa and buckwheat may serve as alternatives to traditional cereals such as rice and bulgur in dietary strategies to reduce cardiometabolic risk in individuals with overweight or obesity.
- The inclusion of quinoa or buckwheat may be a beneficial strategy to increase dietary diversity, however; further studies are needed to determine the most effective dietary patterns and recommendations to observe cardiometabolic health effects.

**Abstract:**

**Background/Objectives**: The study aimed to assess cardiometabolic effects of two pseudocereals, quinoa and buckwheat, comparing them to the effects of rice and bulgur in adults with overweight or obesity. **Methods**: This study was conducted on 148 adults with overweight and obesity, aged between 25 and 45 years, consuming 40 g/d quinoa, buckwheat, rice, or bulgur for 4 weeks. Anthropometrical measurements were taken, and dietary intake was assessed; blood pressure and biochemical parameters were examined. Incremental areas under the curve (iAUC) for glucose and insulin were calculated. Subgroup analyses were conducted considering sex. **Results**: The changes in biochemical and anthropometrical measurements did not differ significantly between groups. However, within-group analysis showed significant changes in many parameters. Among anthropometrical measurements, fat mass declined significantly within quinoa and buckwheat groups (*p* < 0.05). Blood pressure decreased significantly in the buckwheat group, mainly in women. Fasting glucose decreased significantly only in men in the quinoa group. An increase in iAUC glucose within the bulgur group but a significant decrease in iAUC insulin among men within the buckwheat group was recorded. Total cholesterol (Total-C) significantly decreased in men but increased in women following quinoa consumption. On the other hand, a decrease was reported in high-density lipoprotein cholesterol (HDL-C) in women following quinoa and buckwheat consumption (*p* < 0.05). **Conclusions**: Compared with the baseline, the most pronounced changes were observed in blood pressure, lipid profile, and body fat mass, suggesting a potential cardiometabolic benefit of quinoa and buckwheat. However, sex-specific cardiometabolic differences may occur following the consumption of pseudocereal or cereal. Further studies are needed for more robust results.

## 1. Introduction

Obesity is a major health issue globally. The type and amount of carbohydrate play a critical role in cardiometabolic mechanisms underlying obesity such as energy imbalance, high insulin, glucose and lipid levels, increased oxidative stress, and low-grade chronic inflammation [1,2]. Hence, the composition and nutritional quality of carbohydrate sources are crucial to alleviating obesity risk factors.

Cereals, especially rice and wheat, are the main carbohydrate source in human nutrition [3]. They are classified in the *Poaceae* family. *Oryza sativa* is the most commonly consumed species of rice. The minimally processed and unpolished rice with the bran and germ largely retained contains higher dietary fiber. However, rice is most commonly consumed as white rice, which undergoes milling and polishing that removes the bran and germ layers [4]. White rice has the lowest protein, fat, and vitamin and mineral content compared to other cereals [5]. Similar to rice, wheat undergoes varying types and degrees of processing, resulting in a diverse range of wheat-based products. Bulgur is one of these products and most commonly consumed in the Middle East and Mediterranean countries. It is produced predominantly from *Triticum durum* through a series of processing steps, including sorting, cleaning, cooking, drying, and cracking wheat grains [6,7]. The dietary fiber content of bulgur is generally higher than rice [8]. It contains thiamine and niacin vitamins and small amounts of minerals such as calcium and iron. Nevertheless, the nutritional content of bulgur depends on wheat used in its production [8,9].

Pseudocereals, including quinoa and buckwheat, which are considered novel foods, have been increasingly consumed in Europe as alternative carbohydrate sources. *Chenopodium quinoa Wild* is a commonly consumed species of quinoa. There are white, black, and red quinoa seeds regarding botanical subspecies of plant [10]. Although white quinoa is commonly consumed, the studies have reported that black and red quinoa have a higher phytochemical content [10,11]. The most widely cultivated and consumed species of buckwheat are *Fagopyrum esculentum Moench* and *Fagopyrum tataricum Gaertner* [12,13]. The nutrient profile of buckwheat is characterized by high dietary fiber and resistant starch, relatively high quantity and quality of protein, rich mineral content, and a high abundance of bioactive compounds, particularly polyphenols [14,15].

Several studies emphasized the nutritional and functional advantages of quinoa and buckwheat compared to rice and wheat [16,17]. Quinoa and buckwheat have higher plant-protein with lysine essential amino acids, dietary fiber, and unsaturated fatty acid content [18,19,20]. They are rich in several vitamins and minerals, including thiamin, folic acid, vitamin E, calcium, and magnesium [19,20]. Furthermore, quinoa includes hydroxyecdysone, rutin, and saponin, while buckwheat includes fagopyritol, rutin, quercetin, and D-chiro-inositol phytochemicals [21,22]. These bioactive compounds have cardiometabolic health-improving effects by regulating glucose and lipid profiles as well as blood pressure.

These nutritional advantages have created a debate on health advantages about quinoa and buckwheat compared to rice and bulgur, a product of wheat. Despite preclinical studies that examined the functional effects of pseudocereals on cardiometabolic health, the number of human studies on this topic is low [23,24,25]. Limited human studies reported that quinoa can provide a significant decline in blood glucose, insulin, and lipid levels as well as body weight [26,27,28]. Similarly, previous studies suggested that buckwheat may improve the lipid profile; however, serum glucose, insulin and glycated hemoglobin levels, and insulin resistance values exerted in a dose-dependent manner [29,30,31,32]. Furthermore, quinoa or buckwheat exhibited some improvements in oxidation and inflammation status compared to traditional cereals [33,34]. Additionally, some studies indicated their roles in the prevention of rapid fluctuations in glucose and insulin levels compared to corn and wheat [35,36,37,38]. On the other hand, some studies failed to show any differences in appetite scores, lipid profiles, and oxidative status following the consumption of pseudocereals or traditional cereals [39,40,41]. Considering the limited human studies and the gap for clinical practice, further research was required to provide a better understanding for their potential effects. This study aimed to clarify cardiometabolic health improving the effects of two pseudocereals, quinoa and buckwheat, by comparing them to two traditional cereals, rice and bulgur, in adults with overweight and obesity. In this context, it was hypothesized primarily that the consumption of quinoa or buckwheat, compared to rice and bulgur, may improve cardiometabolic risk indices such as body weight, blood glucose, insulin, and lipid levels as well as blood pressure in people with overweight and obesity.

## 2. Materials and Methods

### 2.1. Study Design

This study was a randomized, active-comparator-controlled, open-label clinical trial in four parallel groups. All procedures were performed in accordance with the Declaration of Helsinki. The study was conducted with the written informed consent of all participants. The study was initially approved by Hacettepe University Clinical Research Committee (KA-19102; 12 May 2023) and registered within the Ministry of Health ethics approval system before the onset of enrolment. Subsequently the study was retrospectively registered on ClinicalTrials.gov (number and date; NCT07513610, 14 March 2026). All outcomes reported in this manuscript were pre-specified in the study protocol approved by the ethics committee prior to participant recruitment and remained unchanged throughout the study. Trial protocol and statistical analysis plan can be accessed on (6 April 2026) https://clinicaltrials.gov/study/NCT07513610.

The findings of study were reported in accordance with Consolidated Standards for Reporting Trials (CONSORT) guideline [42]. CONSORT checklist was given in Supplementary Material Table S1. Blood glucose, insulin, lipids levels, iAUC values, blood pressure, Body Mass Index (BMI), and body fat mass were the primary outcome of this trial. Dietary intake was accepted as the secondary outcome. Outcomes were evaluated using both within-group and between-group comparisons.

For the sample size calculation, the results of a previous study, showing 20.53% decrease in LDL-cholesterol level (152.65 ± 39.97 vs. 121.31 ± 43.11 *p* < 0.001), were used (Cohen’s d= 0.75; effect size of f = 0.35) [27]. The calculation based on 90% statistical power and a Type I error rate of 5% (α = 0.05) showed that minimum of 30 participants per group was required, making a total sample size of 120 people with overweight or obesity.

The randomization procedure was performed by an independent researcher using PASS version 11 software (NCSS, Kaysville, UT, USA) [43]. Participants allocated sequential numbering in four groups (1:1:1:1) according to block randomization list. Participants did not have information about randomization sequence. However, complete allocation concealment was not applicable because of the nature of dietary interventional studies. Baseline analyses were conducted to prevent selection bias.

### 2.2. Participants

Adults with overweight and obesity recruited from Department of Endocrinology and Metabolism at Hacettepe University Hospitals in Ankara, Türkiye, between January 2024 and February 2025. The inclusion criteria were as follows: aged between 25 and 45 years, BMI value between 25.0 and 35.0 kg/m^2^, and not taking any medication or supplementation that affect body weight or cardiometabolic parameters. Exclusion criteria were as follows: having a diagnosis of chronic cardiometabolic disease (such as diabetes mellitus, liver disease, kidney disease, etc.) and/or psychiatric illness; taking antibiotics and/or probiotics and/or prebiotics in last three months; having food allergies and/or food intolerances; being in pregnancy, lactation, or postmenopausal period; having a special diet to reduce body weight in the last 6 months; and being unable to attend weekly visits to research center. In case of medication or supplements use or starting a specific diet that can affect body weight, lipid and/or glucose profile, and blood pressure, the participant was excluded from the study.

**Figure 1:**
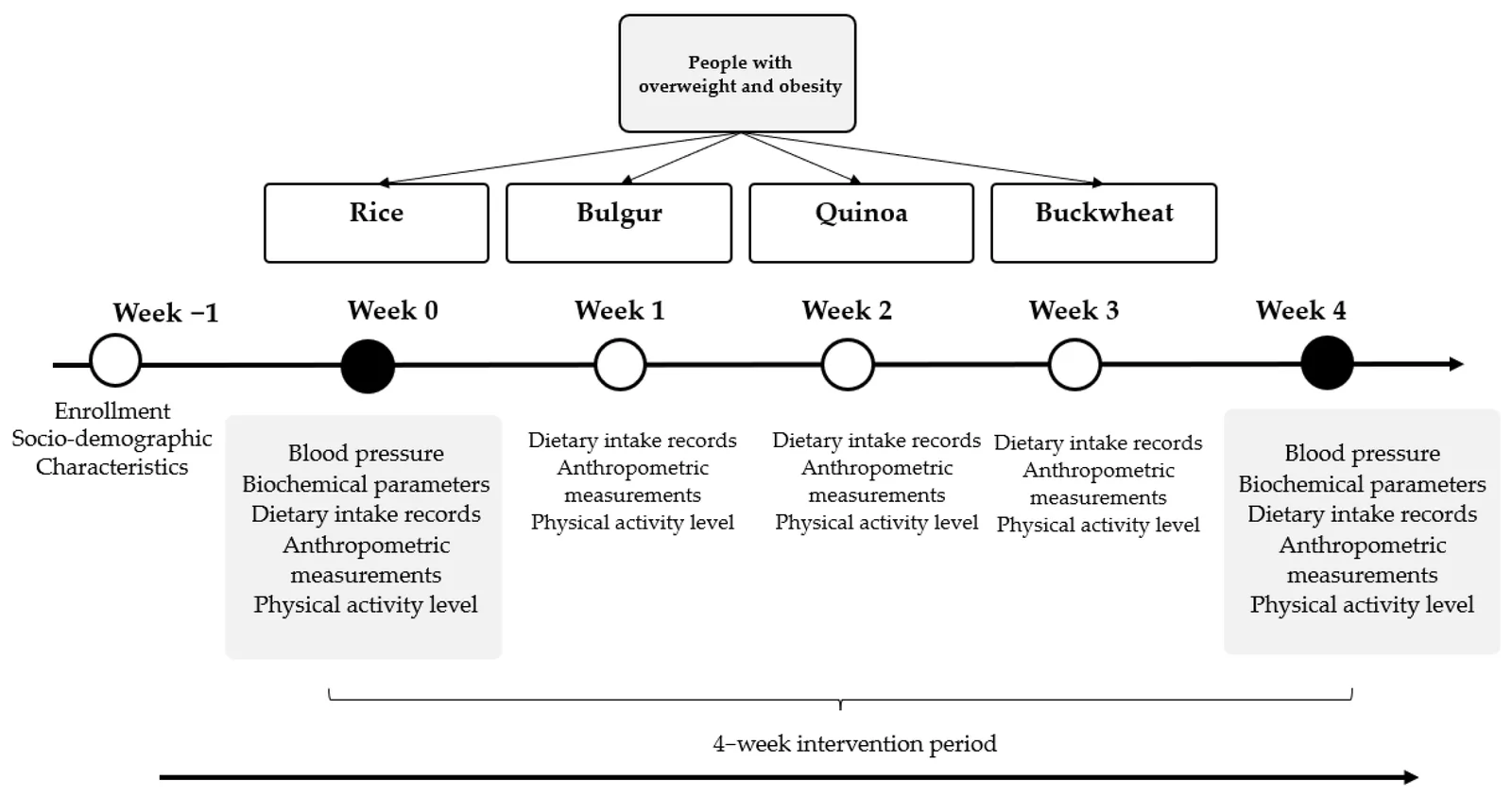
Experimental design of the study.

Figure 1 shows the experimental design of the study. After the screening of participants in terms of inclusion and exclusion criteria by research endocrinologist and dietitian at the first meeting (Week −1 [W−1]), volunteers were enrolled and evaluated in terms of criteria. In second meeting (Week 0, [W0]), participants were included in quinoa, buckwheat, bulgur, or rice group according to randomization list and started four-week intervention period. During the study period, a total of six visits were conducted with the volunteers by research dietitians and/or endocrinologists. Three of these visits (Week −1, 0, and 4 [W−1, W0 and W4]) were held at both the Department of Endocrinology and Metabolism Unit and Department of Nutrition and Dietetics, while the remaining three visits (Week 1, 2, 3 [W1, W2 and W3]) were conducted only at Department of Nutrition and Dietetics.

### 2.3. Test Foods and Instruction for Preparation and Consumption

All pseudocereals/cereals used in the study were provided from a commercial local manufacturer (Duru Gida, Karaman, Türkiye). Rice was white rice, also locally known as osmancik rice (*Oryza sativa L*); bulgur was coarse bulgur (*Triticum durum*). Quinoa was red quinoa (*Chenopodium quinoa*), and buckwheat was common buckwheat (*Fagopyrium Esculentum Moench*).

A comprehensive review of the previous studies showed that the amounts of pseudocereals or cereals consumed in human intervention studies ranged from 15 g to 300 g and administered in various forms such as bread, pasta, porridge, or snacks [20,22,44]. Although there is no established recommendation for the daily consumption of pseudocereals, previous studies have suggested that daily intakes of 25–100 g may be associated with potential benefits for cardiometabolic health [28,45,46]. Therefore, 40 g/day pseudocereals or cereals were selected as an effective and practically achievable amount for incorporation into the habitual diet as a whole food. The nutritional compositions of cereals and pseudocereals (per 100 g in raw) were presented in Table 1 [47].

Pseudocereals/cereals in 40 g were weighed by a kitchen scale (Ideenwelt, Burgvedel, Germany) and packed into transparent Ziplock bags. Eight packages (seven packages for each day of week and one for extra) were given to participants on weekly visits. The preparation methods, including washing, soaking, and cooking, may change the structure of pseudocereal/cereals grain as well as their nutrient profile [48,49,50]. Furthermore, cooking method, including time and temperature, may result in the formation of by-products and alterations in nutritional value of pseudocereals/cereals [9,51]. Correspondingly, these variations in preparation and cooking of pseudocereals/cereals may change their digestion, absorption, and metabolism in the body. Steaming and boiling methods were mainly used in clinical studies conducted with pseudocereals [28,32,45,52] and traditional cereals [53,54,55]. Following a comprehensive review of these previous studies, boiling was selected as the cooking method for this study. After preliminary cooking trials evaluating cooking time, temperature, and water volume, the standardized preparation method was established as cooking each 40 g portion of quinoa, buckwheat, bulgur, or rice in approximately two volumes of water over low heat for 15–20 min until the water was fully absorbed. All participants were instructed to prepare the test foods according to this standardized procedure, and this information was also provided in written. They were also asked to consume the test foods prepared according to this procedure as a part of their main or snack meals, whichever they prefer.

Participants were instructed to not consume buckwheat, quinoa, bulgur, or rice more than provided for each intervention group. Also, except for the pseudocereal/cereal supplementation, they were instructed to maintain their usual dietary habits throughout the study. In order to evaluate participants’ compliance with the study protocol, dietary intake and adherence to the dietary intervention were assessed in detail during weekly face-to-face meetings with the research dietitian throughout the study. These weekly meetings included reviewing the study diary and checklist documenting test food consumptions, checking returned empty or partially consumed pseudocereal/cereal packages, collecting 24 h dietary recalls, and systematically assessing the consumption pattern of all test foods. A total of 10 participants who did not adequately adhere to the dietary intervention were withdrawn from the study.

### 2.4. General Characteristics and Lifestyle Behaviors

General characteristics, including education, health information, medication, smoking, and alcohol consumption, were recorded by research dietitian at W−1. Physical activity type and duration were recorded in every weekly visit, and physical activity level (PAL) was calculated to observe if there were any alterations in physical activity levels of participants. In order to minimize confounding variables, participants were instructed not to make any changes in their routine diets and physical activity levels.

### 2.5. Dietary Assessment

Study diary was provided to each participant at W−1 to record their food intake during the study period. For providing detailed information on participants’ routine diet, they were asked to self-record a five-day food consumption from W−1 to W0. They were also asked to record any side effects that they experienced following the consumption of pseudocereal/cereal in study diary. During the intervention period, dietary intake was assessed using 24 h recall method by a research dietitian in weekly visits. A guideline including photographs of foods items was used to decide the portion sizes. Energy and nutrient intakes were calculated by using the BeBIS Nutrition Information System Software (BeBIS Version 6).

### 2.6. Anthropometric Measurements

Height was measured using a stadiometer (Seca 220, SECA, Hamburg, Germany). Body weight was measured, and body composition was analyzed using segmental body composition analyzer (Tanita MC-980 MA, Tanita Corporation, Tokyo, Japan) by bioelectrical impedance analysis method. The measurements provided fat mass and fat-free mass values in percentage or kilograms. Body mass index (BMI) was calculated as weight in kilograms divided by height in meters squared (kg/m^2^). Waist circumference was measured by a tape (Maxi, Ankara, Türkiye) at the midpoint between the lowest rib and the iliac crest. Hip circumference was measured with a tape at the widest point of the hips. All anthropometrical measurements were taken and assessed according to World Health Organization instructions [56].

### 2.7. Blood Pressure and Biochemical Assessment

Blood glucose, insulin, and lipid levels as well as blood pressure were among the primary hypothesis of this trial. Systolic blood pressure (SBP) and diastolic blood pressure (DBP) were measured at the right arm by using sphygmomanometer (Galena, Istanbul, Türkiye) and stethoscope (Galena, Istanbul, Türkiye). Venous blood samples were collected after 8–12 h fasting and centrifuged at 3000 rpm +4 °C for 10 min immediately. Blood lipid profiles, including triglycerides (TG); HDL-cholesterol (HDL-C); LDL-cholesterol (LDL-C); total cholesterol (Total-C); glucose; insulin levels and HOMA-IR values; liver function tests, including alanine transaminase (ALT) and aspartate transaminase (AST); gamma-glutamyl transferase (GGT) as well as C-reactive protein (CRP); uric acids; and fructosamine levels, were analyzed. For the analysis of oxidative and inflammatory indicators, including malondialdehyde (MDA), interleukin-6 (IL-6), tumor necrosis factor-α (TNF-α), and adiponectin levels, fasting blood serums were stored under −80 °C until the analysis with ELISA method by means of ELISA kits (Elabscience, Houston, TX, USA) in accordance with kit protocols. Additionally, 75 g oral glucose tolerance test (OGTT) was performed before and after the intervention period to assess the postprandial glucose and insulin levels. In OGTT procedure, following drawing of fasting blood samples, the participants were requested to consume a 250 mL dextrose solution in 30% concentration within a few minutes without delay. During the OGTT, participants did not consume any food or drinks and were kept in a sitting position in hospital settings under supervision of the research dietitian. Subsequently, 10 mL blood samples were collected at 30, 60, 90, and 120 minutes, and postprandial insulin and glucose levels were assessed. Biochemical markers were analyzed using routine methods at Hacettepe University Hospitals Biochemistry Laboratory. Blood glucose and insulin fluctuations were evaluated by creating a line graph and calculating incremental area under curve (iAUC) by using following equations according to trapezoid method by Wolever et al. [57].

iAUC equation for blood glucose levels:

iAUC glucose= [(0 + (glucose 30.min− glucose 0.min))/2]×30+[((glucose 30.min− glucose 0.min)+ (glucose 60.min− glucose 0.min))/2]× 30 + [((glucose 60.min− glucose 0.min)+ (glucose 90.min− glucose 0.min))/2]×30 + [((glucose 90.min− glucose 0.min)+ (glucose 120.min− glucose 0.min))/2]× 30

iAUC equation for blood insulin levels:

iAUC insulin = [(0 + (insulin 30.min− insulin 0.min))/2]× 30 + [((insulin 30.min− insulin 0.min)+ (insulin 60.min− insulin 0.min))/2]×30 + [((insulin 60.min− insulin 0.min)+ (insulin 90.min− insulin 0.min))/2]× 30 + [((insulin 90.min− insulin 0.min)+(insulin 120.min− insulin 0.min))/2]× 30 

[57]. Measurements of blood pressure and biochemical parameters were taken in same procedure at W0 and W4. Anthropometrical measurements and dietary assessments were taken on weekly visits.

### 2.8. Statistical Analysis

Statistical analyses of the study were performed using the Statistical Package for the Social Sciences, SPSS version 24.0 (SPSS INC., Chicago, IL, USA). In descriptive analyses, Pearson’s chi-square test and Fisher’s chi-square test were used to analyze differences between groups for categorical variables. Shapiro–Wilk test was used to check for normal distribution of data. Based on Shapiro–Wilk test results, Mann Whitney-U, Kruskal–Wallis, or Wilcoxon tests were used to compare data that did not show normal distribution for intergroup and intragroup comparisons. All data were analyzed within female and male subgroups to evaluate possible sex-specific effects of intervention. When there was a statistically significant difference, pairwise post hoc comparisons were applied using Dunn’s test with Bonferroni correction. Following analysis, data were reported as mean and standard deviation values or the median and minimum–maximum value for continuous variables. All statistical analyses were conducted under 95% confidence interval, and *p*-value < 0.05 was considered statistically significant.

## 3. Results

A total of 151 individuals enrolled in this study. However, three participants changed their consent and declined to participate in the study after enrollment. Consequently, 148 individuals were randomized in the study groups.

**Figure 2:**
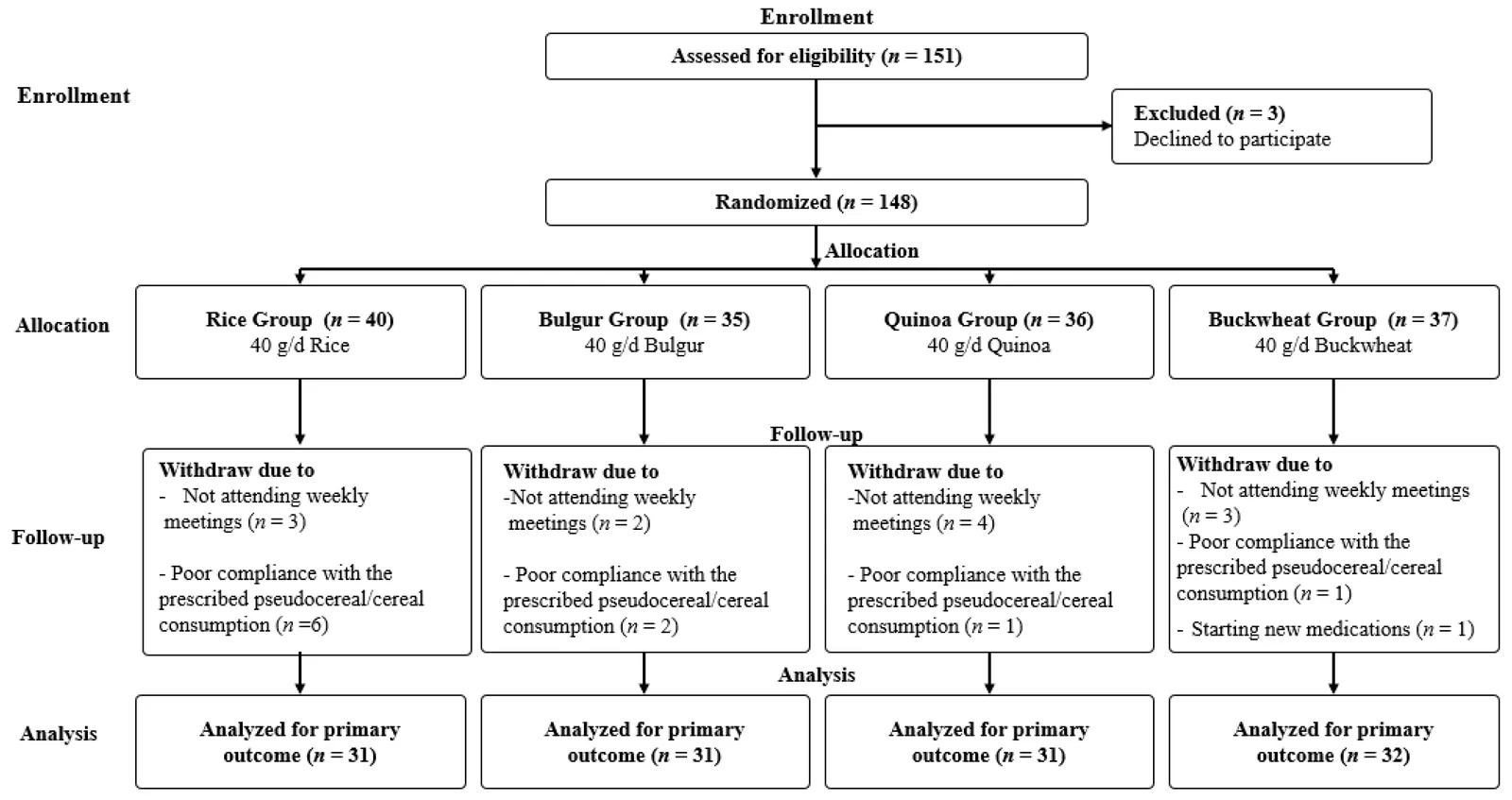
CONSORT flow diagram.

In the follow-up period, 23 individuals withdrew from the study due to not attending weekly meetings, poor compliance with the prescribed pseudocereal/cereal consumption (<6 times/week), and starting a new medication that may affect the outcomes. Hence, 125 volunteers (male = 26; female = 99) completed the study. Participants who completed all study procedures were included in the final analysis, distributed across four groups as rice (*n* = 31; female = 21, male = 10), bulgur (*n* = 31; female = 26, male = 5), quinoa (*n* = 31; female = 26, male = 5), and buckwheat (*n* = 32; female = 26, male = 6). Flow diagram was given in Figure 2.

### 3.1. Baseline Characteristics

The distribution of participant characteristics, including age (between 25–45 years), sex, education level (mainly high school and university education in each group), and health status, was similar across the groups (*p* > 0.05). The reported diagnosed diseases were asthma, gastritis, varicose veins, migraine, and scoliosis. Likewise, there were no significant differences in smoking and alcohol consumption patterns as well as physical activity level (mainly sedentary) between the groups (*p* > 0.05). Table 2 shows the baseline characteristics of the participants.

### 3.2. Cardiometabolic Markers

Table 3 indicates the results of cardiometabolic parameters representing glucolipid metabolism, liver functions, oxidation and inflammation parameters in fasting blood serum, and blood pressure. There were no significant differences in biochemical parameters between the groups (*p* > 0.05) except for iAUC glucose levels before the intervention in men (*p* < 0.05). SBP and DBP levels significantly decreased in women in the buckwheat groups (*p* < 0.05).

Quinoa consumption provided a significant decrease in total cholesterol levels among men while an increase was reported among women (*p* < 0.05). LDL-C decreased significantly in men only in the bulgur group (*p* < 0.05). On the other hand, HDL-C levels decreased in women in both quinoa and buckwheat groups (*p* < 0.05).

Biochemical parameters for inflammation or oxidation status (IL-6, TNF-α, CRP, and MDA), liver functions tests (ALT, AST and GGT), and uric acid did not show any significant differences (*p* > 0.05). Serum CRP levels decreased significantly in both sexes in the rice group, only in males in the quinoa group, and only in women in the bulgur group (*p* < 0.05 for each).

**Figure 3:**
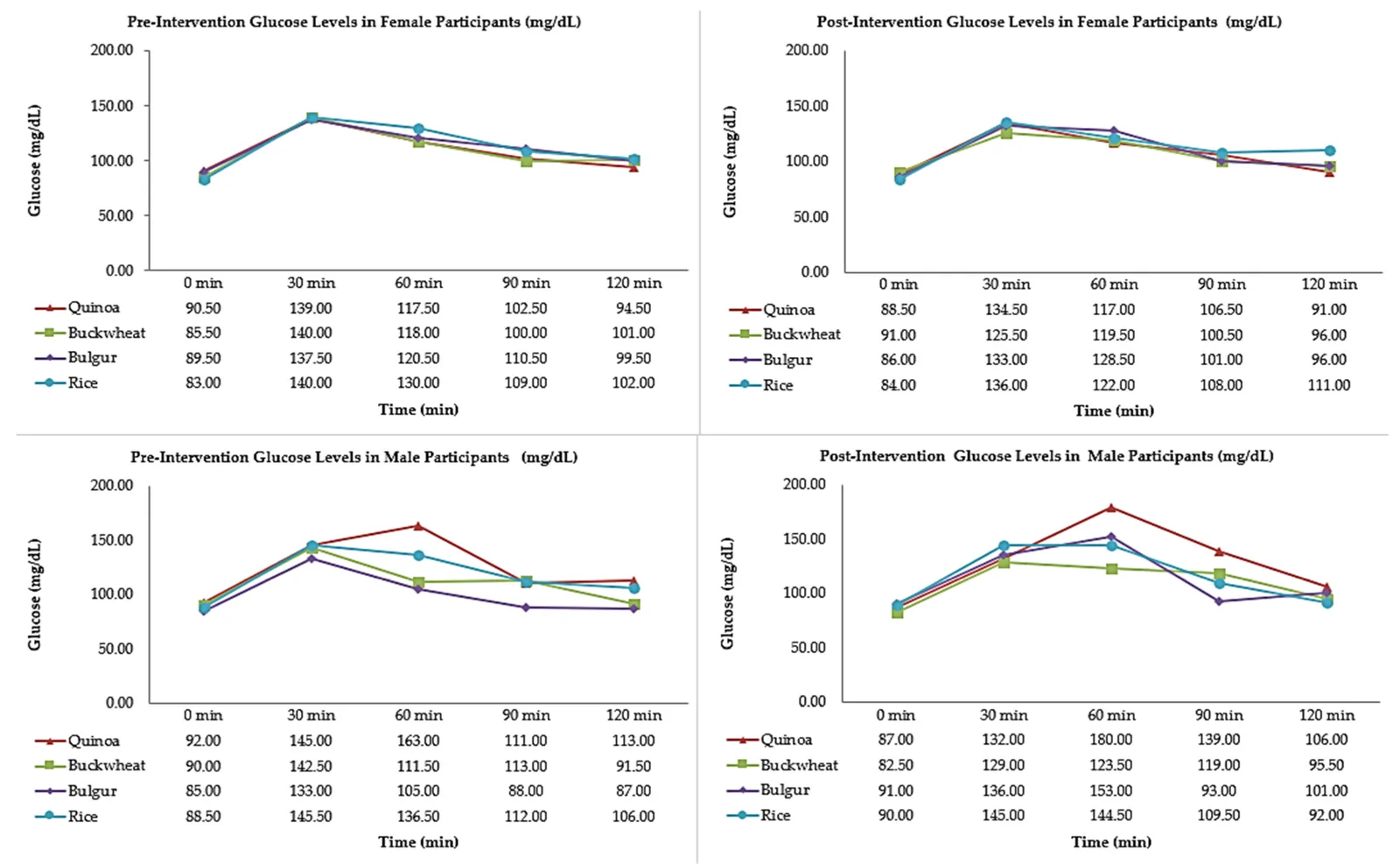
Fluctuations in pre- and postprandial glucose levels. Data were presented as descriptive median values of glucose levels and depicted as line graphs for glucose levels in pre- and post-intervention period. Statistical comparisons were not performed.

**Figure 4:**
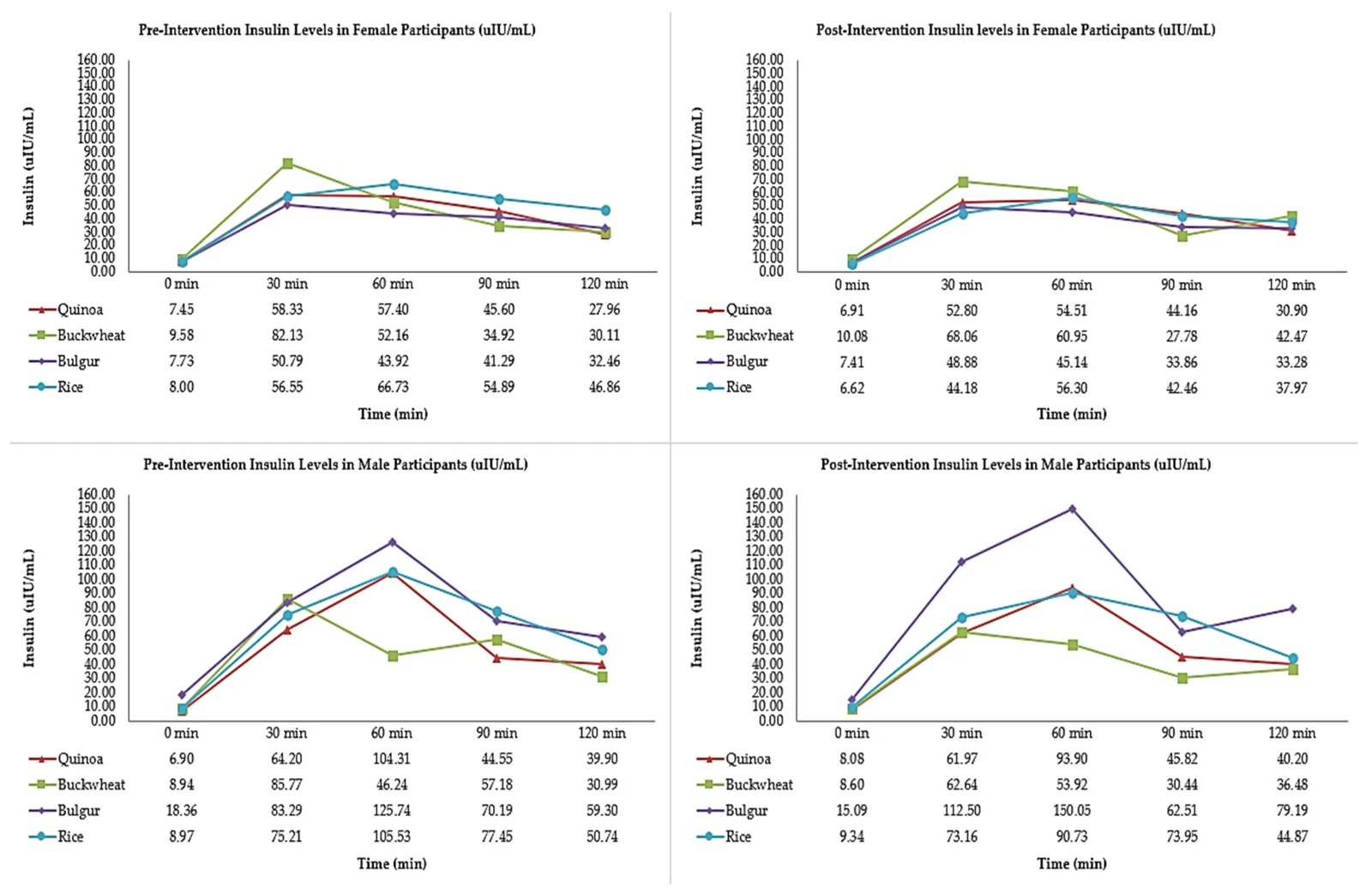
Fluctuations in pre- and postprandial insulin levels. Data were presented as descriptive median values of insulin levels and depicted as line graphs for insulin levels in pre- and post-intervention period. Statistical comparisons were not performed.

Fasting glucose levels decreased significantly in men within both the quinoa and buckwheat groups, while it increased significantly in women within the buckwheat group (*p* < 0.05). Fasting insulin and HOMA-IR levels decreased significantly in females within the rice group (*p* < 0.05). Figure 3 and Figure 4 reflect fluctuations in blood glucose and insulin levels for each group before and after the 4-week intervention period. Values of the incremental area under the curve (iAUC) glucose and insulin values, used to evaluate glycemic regulation, were given in Table 4. Although there was a significant difference in pre-intervention period between groups in males (*p* < 0.05), the difference was not observed in the post-intervention period between groups (*p* > 0.05). iAUC glucose levels increased significantly in males within the bulgur group following the intervention (*p* < 0.05). After the intervention, iAUC insulin levels significantly decreased in men within the buckwheat group (*p* < 0.05). The between-group analysis revealed no significant change in any of the other blood parameters (*p* > 0.05).

### 3.3. Anthropometrical Measurements

Comparison of the effects of pseudocereals and cereals on anthropometrical measurements were given in Table 5. Considering the study population and the primary hypothesis of the study, it was focused on cardiometabolic health-related anthropometric outcomes such as body weight, BMI, waist/hip ratio, and body fat percentage. Body weight and BMI reduced within both sexes in all groups; however, the reductions were statistically significant in rice, bulgur, and buckwheat groups (*p* < 0.05). The analysis of body composition showed that fat mass (%) decreased significantly in the quinoa and buckwheat groups regardless of sex (*p* < 0.05). No significant difference was recorded in waist/hip ratio and fat-free mass following the intervention (*p* > 0.05).

### 3.4. Dietary Intakes

Table 6 indicates dietary energy and nutrient intakes of the groups. Dietary energy and nutrient intakes were similar in each group at the baseline (*p* > 0.05). Energy intakes significantly decreased during the intervention in male participants except the buckwheat group (*p* < 0.05). In female participants, energy intake significantly decreased in the rice group while increasing in the buckwheat group (*p* < 0.05 for each).

The findings of macronutrient intake exerted different tendencies both within and between pseudocereal/cereal groups. Carbohydrate intake increased significantly in men in the rice group (*p* < 0.05). Protein intake increased significantly in men in the bulgur group and in women in the quinoa group (*p* < 0.05). Fat intake significantly decreased in men in all groups except the quinoa group (*p* < 0.05).

When between-group differences were examined, dietary carbohydrate and protein intakes changed significantly among female participants in the post-intervention period (*p* < 0.05). Post hoc pairwise comparisons indicated that differences in carbohydrate intake came from the buckwheat and rice groups, while the difference in protein intakes was between the rice and quinoa groups.

### 3.5. Adverse Events

Notably, in the first week of the intervention period, three participants in the rice group, two participants in the quinoa group, two participants in the bulgur group, and one participant in the buckwheat group reported mild gastrointestinal symptoms such as constipation, diarrhea, and bloating. However, the symptoms were well tolerated, and severe adverse effects were not experienced.

## 4. Discussion

This study was conducted to investigate the cardiometabolic effects of two pseudocereals, quinoa and buckwheat, compared to the effects of two traditional cereals, rice and bulgur. The findings of this study supported the hypothesis that buckwheat can lower blood pressure. In addition, the potential of quinoa and buckwheat to reduce body fat was confirmed in this study. However, between-group comparisons showed the only advantage of buckwheat and quinoa in decreasing carbohydrate intake and increasing protein intake, respectively.

The comparable baseline characteristics of groups, especially age, sex, BMI, physical activity level, dietary intake, and other variables, allowed us to attribute the results specifically to pseudocereal/cereal consumption. The significant change in blood pressure among the female population within the buckwheat group was pronounced. Buckwheat is rich in rutin and quercetin and contributes to the inhibition of ACE activity, endothelial functions, and vasodilation [44]. Considering the findings of this study and the previous literature [5,7], it can be stated that buckwheat may have strong potential to regulate blood pressure. The larger number of male participants may have increased the likelihood of detecting significant changes in men.

The phytosterol and saponin content of quinoa may be responsible for the decrease in Total-C and HDL- C levels by inhibiting cholesterol absorption in male and female participants, respectively. Particularly, 20-Hydroxyecdysone, a kind of phytosterol, is found in quinoa and has the potential to compete with cholesterol during the intestinal lipid absorption and to increase the bile acid and fecal cholesterol excretion [25,58]. A meta-analysis conducted with quinoa showed that quinoa may significantly decrease Total-C levels [59]. Atefi et al. reported that quinoa consumption does not significantly change HDL-C levels, suggesting changes in HDL-C levels may be related to modifications in the type of fat in habitual diet over a longer time [60]. Although quinoa was associated with significant reductions in total cholesterol in the male population, this improvement did not reach the statistical significance level between cereal/pseudocereal groups. The limited sample size within each sex subgroup may be related to these findings. Moreover, it should be noted that LDL and HDL particle size are more crucial indicators for cardiometabolic risk [61]. However, LDL and HDL particle size are evaluated by advanced analytical methods, and it was not feasible to assess in this study due to constraints of the study settings and conditions. Previous studies reported that quinoa or buckwheat consumption led to improvements in serum TG, LDL-C, Total-C, and HDL-C levels among various populations, including postmenopausal women and individuals with diabetes mellitus or hypercholesterolemia [26,32,45,62]. Based on these literature findings, the effects of pseudocereals on lipid profile warrant further investigations.

Evaluating iAUC glucose and insulin values together provides clearer insight into glycemic regulation. In male participants, consuming buckwheat resulted in a significant decrease in iAUC insulin, whereas iAUC glucose showed a non-significant increase. This situation suggests that the reduced insulin levels in the buckwheat group may reflect a compensatory response to provide glucose homeostasis. Although an increase in iAUC glucose was observed in the buckwheat group, the concomitant reduction in HOMA-IR levels in the male population consuming buckwheat supported the role of buckwheat in improving insulin sensitivity. In another study, buckwheat bread prepared with 50% buckwheat flour decreased postprandial glucose levels and iAUC in healthy adults [35]. Buckwheat crackers regulated incretin hormone levels but did not significantly change the postprandial glucose or insulin levels in individuals with type 2 diabetes [37]. The role of buckwheat on glucose and insulin levels depends on the content of dietary fiber and phenolic compounds, which may delay carbohydrate digestion, prevent glycemic fluctuations, and regulate glucose and insulin metabolism [39]. However, two studies that compared corn bread and buckwheat bread reported that corn bread unexpectedly led to a lower AUC for glucose [63,64]. Although Cesur et al. found no significant difference in total AUC for glucose (0–120 min) between corn bread and buckwheat bread, some significant differences were observed at certain time points. Moreover, buckwheat bread caused higher glucose responses despite the higher dietary fiber of buckwheat. The researchers explained this unexpected change with the high amylose content of corn bread [65].

Previous studies showed that quinoa and buckwheat may support a decrease in oxidative and inflammatory markers such as MDA, TNF-α, and IL-8 [30,34]. Similarly, De Carvalho et al. found that the consumption of 25 g/d quinoa flakes increased glutathione levels [33]. Nevertheless, there were no significant changes in oxidation and inflammation parameters in this study. This might be explained by the low baseline levels of oxidation and inflammation parameters. Furthermore, long-term and comprehensive changes in diet may provide more certain results in oxidation and inflammation status.

Although rice, bulgur, and buckwheat groups showed a statistically significant decrease in body weight and BMI values compared to baseline in this study, these changes did not reach the statistically significant level in between-group comparisons. This situation means that the magnitudes of the decreases were similar across the groups. The absence of significant differences in body weight and BMI between groups suggested that quinoa and buckwheat did not have a significant advantage over rice and bulgur with respect to BMI. The changes observed in body composition support that quinoa and buckwheat consumption exerted their effects on fat mass independent from sex-specific factors in people with overweight and obesity. Furthermore, the absence of baseline differences between the groups, together with the use of randomization, likely minimized the potential influence of this effect.

According to previous studies [62,65,66], the reduction of fat mass within quinoa and buckwheat groups observed in this study may be explained by the high-quality plant protein and bioactive compounds such as rutin and saponins found in pseudocereals. Plant protein composition in quinoa and buckwheat may prolong satiety duration, decreasing appetite and food intake. Moreover, polyphenols may increase thermogenesis and lipid oxidation [67]. Briefly, these functional properties of pseudocereals may affect body composition by improving adipose tissue metabolism.

In the present study, the observed changes in energy and macronutrient intakes exhibited a heterogeneous result according to sex and pseudocereal/cereal group-based analysis. However, the significant sex-based differences within the quinoa and bulgur groups as well as a significant difference in the female population between groups during the intervention showed that cereal or pseudocereal preferences can shape the macronutrient composition of the diet. Nevertheless, further research is needed to confirm these findings.

In contrast to the above studies that investigate the effects of pseudocereals on type 2 diabetes and cardiovascular diseases, this study was conducted on people with overweight and obesity. It is well established that individuals with less favorable metabolic parameters at baseline may exhibit more pronounced responses to the dietary interventions than those with values closer to the normal range [68,69]. Furthermore, the concept of “metabolically healthy obesity”, referring to individuals with obesity but without overt metabolic abnormalities, has provided a different perspective on this issue [70]. The reason for non-significant findings in this study may be associated with mild dysfunctions in the study population compared to other studies conducted on disease conditions. On the other hand, this situation strengthened the study results and provided attributable findings to the pseudocereal/cereal intervention by decreasing confounding factors such as the effects of baseline impaired metabolism or pharmacological treatment because these confounding factors may mask or overestimate the effect of intervention. The other strength of this study is that it has a randomized controlled design and similar individual characteristics at baseline. The use of active comparator groups represented a methodological strength of the study, as it allowed for the assessment of the relative effects of quinoa and buckwheat compared with commonly consumed rice and bulgur rather than only demonstrating their efficacy against non-intervention control. The results of the study offer new data for comparisons of two novel pseudocereal and two common conventional cereals on the cardiometabolic system in people with overweight and obesity.

The limitations of this study can be specified as follows: Firstly, the study has a sex-stratified approach without any additional adjustment. The sex-stratified approach provides an evaluation of sex-specific pairwise comparisons separately. However, this situation has relatively decreased the number of participants in subgroups. Notably, the small number of male participants in subgroups may restrict the generalizability of findings in the male population. There is a Type I error risk in studies with a small sample size. Therefore, future studies conducted with larger male population are required to validate findings. Secondly, this study focused on the potential effects of pseudocereal/cereal when consumed as a whole food within the diet, rather than their carbohydrate, energy, or specific nutrient content. However, the slight difference in the energy (ranging from 1.60–11.60 kcal) and carbohydrate content (ranging from 1.64–6.40 g) between 40 g of pseudocereals or cereals may be considered as another limitation. On the other hand, these differences may be negligible in such studies where energy intake is not restricted. Thirdly, the intervention duration may be considered as another limitation because 4 weeks may be too short to detect differences in some of the liver function and inflammation parameters. Based on the previous literature as well as considering individuals’ compliance with protocol and the risk of withdrawal from the study, the intervention duration was planned for 4 weeks. Dropouts were the most significant challenge in follow-up intervention studies. Nevertheless, longer intervention periods may allow for more significant changes. Finally, another limitation of this study was that study registration on ClinicalTrials.gov was performed retrospectively after participant recruitment had commenced. Although all outcomes analyzed and presented in this manuscript were defined a priori and remained unchanged, the retrospective registration may limit the perceived transparency of outcome reporting.

## 5. Conclusions

The findings of this study provided evidence on the potential benefits of quinoa and buckwheat (especially in blood pressure and body fat mass) regarding sex-specific differences on cardiometabolic health in adults with overweight and obesity. It suggested that increasing diet diversity through the inclusion of pseudocereals, rather than relying on a single staple cereal, may be a part of sustainable healthy diet strategy. While the findings of the current study were promising, further human studies in larger populations with longer intervention periods are still warranted.

## Figures and Tables

**Table 1:** Energy and macronutrients composition of rice, bulgur, quinoa, and buckwheat.

Cereals/ Pseudocereals (100 g, Raw)	Energy (Kcal)	Carbohydrates (g)	Dietary Fiber (g)	Protein (g)	Fat (g)
Rice	359.00	80.00	1.00	7.00	1.00
Bulgur	372.00	75.90	11.70	11.80	2.40
Quinoa	368.00	64.00	7.00	14.00	6.00
Buckwheat	343.00	71.50	10.00	13.00	3.40

**Table 2:** Baseline characteristics of participants.

	Rice (*n* = 31)	Bulgur (*n* = 31)			Quinoa (*n* = 31)		Buckwheat (*n* = 32)		*p* ^between groups^
	Median (Min–Max)	Median (Min–Max)			Median (Min–Max)		Median (Min–Max)		
**Age (years)**	35.00 (25.00–45.00)	36.00 (25.00–45.00)			37.00 (26.00–44.00)		35.00 (25.00–45.00)		0.702
	***n***	**%**	***n***	**%**	***n***	**%**	***n***	**%**	
**Sex**									0.403
Female	21	67.80	26	83.90	26	83.90	26	81.20	
Male	10	32.20	5	16.10	5	16.10	6	18.80	
**Education**									0.263
Literate	**-**	**-**	1	3.20	**-**	**-**	**-**	-	
Primary School	1	3.20	1	3.20	3	9.70	2	6.30	
Middle School	3	9.70	4	12.90	4	12.90	4	12.50	
High School	12	38.70	13	41.90	9	29.00	11	34.30	
University	15	48.40	12	38.80	15	48.40	15	46.90	
**Diagnosed Disease**									0.081
Yes	4	13. 00	4	13.00	1	3.20	-	-	
No	27	87. 00	27	87.00	30	96.80	32	100.00	
**Smoking**									0.261
No	22	71.00	25	80.60	21	67.80	23	71.90	
Yes, regularly	8	25.80	4	12.90	5	16.10	3	9.40	
Yes, sometimes	1	3.20	2	6.50	5	16.10	6	18.70	
**Alcohol consumption**									0.974
Yes	3	9.70	3	9.70	2	6.50	4	12.50	
No	28	90.30	28	90.30	29	93.50	28	87.50	
**Physical Activity Level**									0.344
Sedentary	28	90.30	28	90.30	27	87.00	26	81.30	
Active	3	9.70	3	9.70	2	6.50	6	18.70	
Very Active	-	-	-	-	2	6.50	-	-	

Data were represented as median (minimum–maximum) for age and number (*n*) and percentage (%) for other variables. Statistically significant *p* values (*p* < 0.05) were indicated as bold. *p* between-groups value for age was obtained by Kruskal–Wallis test. *p* between-groups values for other variables were obtained by Fisher exact test. (-) shows no cases observed.

**Table 3:** Blood parameters and pressures pre- and post-intervention period.

		Pre-Intervention				Post-Intervention					
	Female (*n* = 99)			Male (*n* = 26)		Female (*n* = 99)		Male (*n* = 26)			
Fasting Glucose (mg/dL)	Groups (F/M)	X ± SD	Median (Min–Max)	X ± SD	Median (Min–Max)	X ± SD	Median (Min–Max)	X ± SD	Median (Min–Max)	*p* ^within (F)^	*p* ^within (M)^
	**Groups** **(F/M)**	84.76 ± 7.70	83.00 (67.00–101.00)	89.50 ± 10.74	88.50 (73.00–108.00)	84.67 ± 6.73	84.00 (72.00–96.00)	89.50 ± 8.40	90.00 (78.00–101.00)	0.762	0.953
	**Rice** **(21/10)**	88.38 ± 10.00	89.50 (66.00–111.00)	92.40 ± 11.72	85.00 (83.00–108.00)	88.73 ± 10.52	86.00 (70.00–119.00)	90.40 ± 11.89	91.00 (76.00–102.00)	0.898	0.465
	**Bulgur** **(26/5)**	90.46 ± 8.46	90.50 (76.00–111.00)	93.40 ± 7.44	92.00 (83.00–102.00)	90.92 ± 9.16	88.50 (76.00–109.00)	87.20 ± 7.56	87.00 (77.00–98.00)	0.893	0.043 *
	**Quinoa** **(26/5)**	86.27 ± 5.72	85.50 (74.00–97.00)	90.00 ± 5.25	90.00 (83.00–98.00)	90.81 ± 8.51	91.00 (72.00–104.00)	82.00 ± 6.07	82.50 (72.00–89.00)	0.007 *	0.027 *
***p*** **^between-groups^**		0.098		0.790		0.068		0.427			
**Fasting Insulin** **(uU/mL)**	**Groups** **(F/M)**	8.83 ± 3.60	8.00 (4.36–16.00)	12.56 ± 7.00	8.96 (6.34–28.98)	7.05 ± 2.63	6.62 (3.30–14.00)	19.34 ± 20.14	9.34 (7.20–68.59)	0.023 *	0.799
	**Rice** **(21/10)**	8.82 ± 4.24	7.73 (2.30–18.77)	16.53 ± 9.60	10.36 (7.33–30.61)	8.76 ± 4.05	7.41 (3.94–22.13)	15.98 ± 10.90	15.09 (5.57–33.94)	0.677	0.500
	**Bulgur** **(26/5)**	8.86 ± 6.29	7.45 (2.60–30.20)	7.18 ± 2.38	6.90 (4.25–10.88)	9.43 ± 6.55	6.91 (2.62–27.90)	7.83 ± 1.35	8.08 (6.20–9.29)	0.716	0.345
	**Quinoa** **(26/5)**	10.40 ± 5.17	9.58 (2.48–27.96)	10.52 ± 7.02	8.94 (2.49–21.60)	11.45 ± 6.65	10.07 (2.12–25.38)	10.95 ± 8.36	8.60 (0.95–23.18)	0.732	0.917
***p*** **^between-groups^**		0.685		0.152		0.070		0.345			
**Fructosamine** **(μmol/L)**	**Groups** **(F/M)**	232.38 ± 38.92	238.00 (132.00–326.00)	243.20 ± 17.22	241.50 (220.00–277.00)	236.10 ±38.61	235.00 (106.00–293.00)	240.80 ± 29.66	243.50 (185.00–280.00)	0.268	0.959
	**Rice** **(21/10)**	231.35 ± 23.27	232.50 (190.00–274.00)	251.60 ± 38.95	258.00 (188.00–293.00)	248.96 ±19.67	245.50 (217.00–292.00)	238.20 ± 37.99	233.00 (190.00–296.00)	0.588	0.225
	**Bulgur** **(26/5)**	228.8 ± 32.90	235.5 (160.0–289.0)	226.23 ± 35.3	230.5 (106.0–308.0)	226.23 ±35.33	230.50 (106.00–308.00)	255.00 ± 5.39	256.00 (246.00–260.00)	0.686	0.686
	**Quinoa** **(26/5)**	238.15 ± 42.88	241.00 (98.00–334.00)	235.17 ± 31.43	229.50 (192.00–272.00)	237.96 ±56.63	257.50 (94.00–302.00)	253.00 ± 24.83	247.00 (229.00–289.00)	0.316	0.249
***p*** **^between-groups^**		0.677		0.700		0.055		0.530			
**HOMA-IR**	**Groups** **(F/M)**	1.87 ± 0.84	1.68 (0.78–3.51)	2.78 ± 1.61	2.12 (1.28–6.66)	1.46 ± 0.67	1.36 (0.19–3.28)	4.14 ± 5.04	2.02 (0.25–16.94)	0.025 *	0.959
	**Rice** **(21/10)**	1.93 ± 0.97	1.66 (0.46–4.00)	3.96 ± 2.73	3.85 (1.50–8.16)	1.92 ± 0.89	1.65 (0.84–4.43)	3.71 ± 2.88	2.99 (1.11–8.55)	0.737	0.345
	**Bulgur** **(26/5)**	2.04 ± 1.64	1.65 (0.51–8.20)	1.68 ± 0.64	1.64 (0.87–2.66)	1.80 ± 1.64	1.42 (0.22–7.10)	1.70 ± 0.40	1.78 (1.27–2.25)	0.551	0.893
	**Quinoa** **(26/5)**	2.24 ± 1.19	2.01 (0.55–6.28)	2.27 ± 1.41	2.03 (0.60–4.43)	2.32 ± 1.89	1.80 (0.20–6.27)	1.93 ± 2.02	1.27 (0.04–4.98)	0.949	0.342
**p ^between-groups^**		0.475		0.343		0.302		0.360			
**Total-C** **(mg/dL)**	**Groups** **(F/M)**	184.24 ± 40.93	168.00 (138.00–307.00)	179.80 ± 28.15	175.00 (133.00–231.00)	182.24 ± 35.38	179.00 (124.00–265.00)	177.10 ± 24.64	176.00 (138.00–218.00)	0.715	0.594
	**Rice** **(21/10)**	193.88 ± 36.83	180.50 (145.00–265.00)	176.40 ± 39.88	167.00 (131.00–240.00)	192.96 ± 44.58	172.50 (136.00–320.00)	166.40 ± 46.64	164.00 (101.00–230.00)	0.909	0.144
	**Bulgur** **(26/5)**	198.58 ± 37.50	192.50 (140.00–289.00)	225.40 ± 67.22	205.00 (151.00–329.00)	186.04 ± 31.35	196.50 (112.00–240.00)	205.20 ± 66.58	188.00 (129.00–309.00)	0.017 *	0.043 *
	**Quinoa** **(26/5)**	188.08 ± 35.77	180.50 (126.00–273.00)	215.83 ± 63.04	202.00 (138.00–297.00)	186.35 ± 38.75	188.00 (112.00–267.00)	218.17 ± 65.29	208.00 (128.00–312.00)	0.517	0.600
***p*** **^between-groups^**		0.434		0.309		0.891		0.359			
**LDL-C** **(mg/dL)**	**Groups** **(F/M)**	113.10 ± 22.69	105.00 (80.00–164.00)	118.70 ± 21.42	116.00 (82.00–155.00)	118.33 ± 29.57	119.00 (64.00–206.00)	118.40 ± 17.86	114.50 (90.00–145.00)	0.147	0.812
	**Rice** **(21/10)**	126.23 ± 29.09	119.50 (88.00–190.00)	110.80 ± 28.60	112.00 (74.00–153.00)	125.27 ± 32.21	116.50 (85.00–215.00)	101.20 ± 34.74	101.00 (53.00–151.00)	0.706	0.042 *
	**Bulgur** **(26/5)**	124.73 ± 27.45	117.00 (86.00–186.00)	149.20 ± 57.19	134.00 (90.00–240.00)	122.27 ± 24.09	124.50 (76.00–166.00)	132.20 ± 45.48	138.00 (81.00–201.00)	0.331	0.080
	**Quinoa** **(26/5)**	118.65 ± 29.40	114.50 (67.00–186.00)	141.67 ± 45.38	130.50 (87.00–198.00)	123.38 ± 30.29	126.50 (63.00–180.00)	148.17 ± 52.09	136.50 (87.00–232.00)	0.106	0.345
***p*** **^between-groups^**		0.371		0.390		0.861		0.391			
**HDL-C** **(mg/dL)**	**Groups** **(F/M)**	52.38 ± 11.82	49.00 (39.00–93.00)	40.60 ± 5.10	40.50 (34.00–50.00)	53.14 ± 11.42	50.00 (40.00–92.00)	40.10 ± 5.43	39.50 (34.00–53.00)	0.820	0.590
	**Rice** **(21/10)**	50.81 ± 6.93	49.50 (41.00–67.00)	48.80 ± 7.63	45.00 (41.00–57.00)	48.69 ± 7.69	48.50 (32.00–65.00)	47.40 ± 8.56	44.00 (39.00–58.00)	0.120	0.141
	**Bulgur** **(26/5)**	52.96 ± 12.65	52.50 (22.00–79.00)	52.40 ± 21.76	46.00 (34.00–85.00)	47.69 ± 11.78	48.00 (24.00–73.00)	50.00 ± 20.72	42.00 (32.00–82.00)	<0.001 *	0.078
	**Quinoa** **(26/5)**	51.69 ± 10.60	50.50 (33.00–71.00)	48.67 ± 8.62	49.50 (34.00–60.00)	49.00 ± 8.80	49.00 (36.00–72.00)	48.33 ± 8.73	52.00 (32.00–55.00)	0.012 *	0.833
***p*** **^between-groups^**		0.819		0.210		0.526		0.267			
**TG** **(mg/dL)**	**Groups** **(F/M)**	96.10 ± 46.68	91.00 (39.00–212.00)	166.80 ± 43.27	170.00 (111.00–226.00)	94.90 ± 38.61	90.00 (42.00–185.00)	171.50 ± 69.91	150.50 (106.00–344.00)	0.793	0.799
	**Rice** **(21/10)**	112.27 ± 51.63	102.50 (50.00–263.00)	128.20 ± 44.05	127.00 (77.00–182.00)	112.77 ± 48.23	101.00 (54.00–199.00)	104.00 ± 41.44	91.00 (50.00–152.00)	0.694	0.223
	**Bulgur** **(26/5)**	102.69 ± 44.94	105.50 (37.00–190.00)	145.20 ± 79.37	158.00 (41.00–227.00)	110.04 ± 54.32	107.50 (42.00–221.00)	164.80 ± 80.68	202.00 (48.00–246.00)	0.068	0.068
	**Quinoa** **(26/5)**	97.23 ± 38.83	89.50 (42.00–167.00)	177.50 ± 111.82	154.00 (61.00–393.00)	101.04 ± 46.25	90.00 (39.00–228.00)	177.33 ± 112.31	172.00 (42.00–366.00)	0.431	0.970
***p*** **^between-groups^**		0.690		0.680		0.652		0.361			
**ALT** **(U/L)**	**Groups** **(F/M)**	17.28 ± 12.71	14.00 (4.80–66.00)	32.00 ± 11.46	30.50 (21.00–57.00)	15.71 ± 4.61	15.00 (9.00–24.00)	30.90 ± 15.60	27.00 (17.00–69.00)	0.948	0.721
	**Rice** **(21/10)**	15.42 ± 5.90	13.00 (9.00–29.00)	34.80 ± 24.89	29.00 (15.00–78.00)	15.96 ± 6.12	13.50 (10.00–34.00)	31.20 ± 20.85	20.00 (15.00–66.00)	0.478	0.273
	**Bulgur** **(26/5)**	21.17±13.34	16.00 (4.20-55.00	31.00 ± 6.82	31.00 (23.00–40.00)	21.97 ± 12.70	20.50 (4.20–61.00)	34.00 ± 8.49	31.00 (25.00–43.00)	0.449	0.144
	**Quinoa** **(26/5)**	19.75 ± 12.60	16.50 (5.10–53.00)	31.17 ± 15.09	26.50 (20.00–61.00)	22.45 ± 19.48	17.00 (5.20–87.00)	28.00 ± 8.81	26.50 (16.00–39.00)	0.735	0.588
***p*** **^between-groups^**		0.429		0.872		0.188		0.625			
**AST** **(U/L)**	**Groups** **(F/M)**	21.86 ± 12.12	19.00 (14.00–72.00)	32.50 ± 21.85	25.50 (22.00–94.00)	19.90 ± 3.94	19.00 (12.00–27.00)	25.40 ± 6.33	25.00 (15.00–37.00)	0.981	0.262
	**Rice** **(21/10)**	18.85 ± 3.57	20.00 (13.00–25.00)	27.40 ± 14.35	22.00 (20.00–53.00)	19.42 ± 4.67	17.50 (12.00–32.00)	25.00 ± 7.84	23.00 (19.00–38.00)	0.540	0.680
	**Bulgur** **(26/5)**	27.27 ± 21.32	20.00 (11.00–105.00)	24.00 ± 3.00	23.00 (21.00–29.00)	23.38 ± 7.52	21.50 (14.00–50.00)	27.40 ± 5.41	27.00 (20.00–35.00)	0.449	0.225
	**Quinoa** **(26/5)**	21.50 ± 5.69	21.00 (12.00–33.00)	24.00 ± 5.80	22.50 (19.00–32.00)	22.42 ± 7.17	19.50 (15.00–43.00)	24.17 ± 2.93	23.50 (21.00–29.00)	0.500	0.916
***p*** **^between-groups^**		0.326		0.336		0.088		0.642			
**GGT** **(U/L)**	**Groups** **(F/M)**	15.48 ± 6.60	14.00 (8.00–34.00)	38.60 ± 13.58	39.00 (21.00–60.00)	14.71 ± 4.23	15.00 (8.00–24.00)	36.90 ± 14.03	40.00 (18.00–55.00)	0.514	0.959
	**Rice** **(21/10)**	16.27 ± 5.90	15.50 (9.00–33.00)	22.60 ± 6.58	21.00 (15.00–32.00)	15.12 ± 4.92	14.50 (9.00–28.00)	44.40 ± 53.23	21.00 (13.00–139.00)	0.053	0.991
	**Bulgur** **(26/5)**	18.88 ± 8.31	16.50 (8.00–37.00)	25.20 ± 6.98	22.00 (20.00–37.00)	19.65 ± 8.33	19.00 (7.00–47.00)	26.80 ± 6.02	29.00 (19.00–33.00)	0.403	0.715
	**Quinoa** **(26/5)**	16.73 ± 8.55	14.00 (9.00–49.00)	32.00 ± 21.65	24.00 (21.00–76.00)	15.54 ± 7.03	12.50 (7.00–39.00)	28.33 ± 18.08	21.50 (19.00–65.00)	0.266	0.136
***p*** **^between-groups^**		0.382		0.052		0.270		0.512			
**Uric Acid** **(mg/dL)**	**Groups** **(F/M)**	5.10±2.00	4.80 (3.40-13.0)	6.47 ± 0.97	6.30 (5.00–7.90)	4.57 ± 0.91	4.20 (3.40–6.60)	6.75 ± 1.72	6.30 (4.60–10.00)	0.120	0.959
	**Rice** **(21/10)**	4.67±1.25	4.50 (3.00-8.40)	6.58 ± 1.02	6.80 (5.20–7.60)	4.58 ± 0.91	4.75 (2.70–6.30)	6.26 ± 0.88	6.30 (5.10–7.50)	0.932	0.588
	**Bulgur** **(26/5)**	4.75±0.82	4.65 (3.80-6.30)	6.56 ± 1.71	5.90 (5.10–9.50)	4.76 ± 0.97	4.40 (3.20–6.90)	7.24 ± 2.04	6.60 (4.80–9.50)	0.898	0.273
	**Quinoa** **(26/5)**	5.10±1.13	5.10 (3.30-7.10)	6.48 ± 1.09	6.05 (5.50–8.30)	5.18 ± 0.99	5.05 (3.50–7.80)	6.93 ± 1.31	6.30 (5.80–8.70)	0.696	0.116
***p*** **^between-groups^**		0.098		0.790		0.097		0.828			
**Adiponectin** **(ng/mL)**	**Rice** **(21/10)**	2.83 ± 1.52	2.64 (0.73–7.07)	2.11 ± 1.28	1.64 (1.00–4.99)	2.91 ± 1.45	2.79 (0.86–5.74)	1.89 ± 1.37	1.53 (0.73–5.43)	0.543	0.285
	**Bulgur** **(26/5)**	3.24 ± 2.19	2.61 (0.69–8.66)	4.14 ± 2.75	5.05 (0.44–7.45)	3.12 ± 2.02	2.54 (0.64–8.54)	2.43 ± 1.85	1.86 (0.84–5.45)	0.264	0.345
	**Quinoa** **(26/5)**	2.73 ± 1.09	2.52 (1.21–5.30)	2.18 ± 0.74	2.02 (1.24–3.15)	3.04 ± 3.50	2.25 (1.11–19.03)	2.39 ± 0.46	2.15 (1.97–3.06)	0.216	0.500
	**Buckwheat (26/6)**	3.83 ± 3.12	3.36 (0.65–17.00)	2.36 ± 1.84	1.75 (0.64–5.82)	3.10 ± 1.47	3.19 (0.51–5.79)	1.94 ± 1.74	1.21 (0.28–5.06)	0.115	0.028 *
***p*** **^between-groups^**		0.396		0.481		0.388		0.346			
**MDA** **(ng/mL)**	**Rice** **(21/10)**	2654.78 ± 980.41	2496.90 (1132.70–4613.70)	2395.98 ± 805.42	2247.00 (1413.90–4298.60)	2996.69± 1592.12	2683.80 (514.20–7169.40)	2481.44 ± 595.29	2292.70 (1714.20–3800.60)	0.455	0.445
	**Bulgur** **(26/5)**	2132.36 ± 927.80	1856.95 (433.25–3995.10)	2662.00 ± 330.20	2717.30 (2214.50–3006.80)	2395.48 ± 925.33	2252.65 (894.52–4823.00)	2814.03 ± 547.52	2577.45 (2226.50–3562.00)	0.341	0.686
	**Quinoa** **(26/5)**	2820.77 ± 1086.42	2550.85 (1185.30–5764.10)	2248.80 ± 1052.50	1755.30 (1280.70–3696.00)	2609.62 ± 924.89	2521.25 (1210.00–5756.40)	2427.10 ± 703.39	2356.50 (1447.40–3242.90)	0.949	0.498
	**Buckwheat (26/6)**	2425.50 ± 1169.11	2348.05 (439.28–5315.90)	1763.82 ± 347.60	1831.75 (1302.10–2199.70)	2427.33± 1057.40	2326.70 (437.96–5299.90)	1937.21 ± 970.89	1713.40 (835.65–3621.80)	0.131	0.463
***p*** **^between-groups^**		0.129		0.088		0.406		0.246			
**CRP** **(mg/L)**	**Rice** **(21/10)**	4.49 ± 3.04	3.10 (0.70–11.30)	4.17 ± 1.79	4.03 (0.70–6.80)	3.63 ± 2.74	2.37 (0.40–9.21)	3.54 ± 1.74	3.45 (0.30–6.00)	0.025 *	0.008 *
	**Bulgur** **(26/5)**	5.72 ± 3.80	5.15 (1.60–18.40)	3.56 ± 2.21	3.70 (0.50–6.12)	4.06 ± 2.83	4.05 (0.50–10.90)	3.53 ± 1.94	3.80 (0.40–5.72)	0.001 *	0.500
	**Quinoa** **(26/5)**	4.39 ± 1.95	4.45 (0.60–9.71)	2.75 ± 1.50	2.72 (1.20–5.10)	4.10 ± 2.15	3.91 (1.00–9.45)	1.82 ± 0.64	1.89 (0.90–2.70)	0.303	0.043 *
	**Buckwheat (26/6)**	3.49 ± 1.59	3.39 (0.50–7.20)	4.13 ± 2.06	4.74 (1.50–6.91)	3.25 ± 1.64	3.58 (0.40–6.50)	3.38 ± 1.89	3.00 (1.77–6.80)	0.054	0.173
***p*** **^between-groups^**		0.151		0.585		0.604		0.217			
**IL-6** **(pg/mL)**	**Rice** **(21/10)**	0.45 ± 0.53	0.35 (0.04–2.43)	0.30 ± 0.19	0.29 (0.18–0.66)	0.53 ± 0.74	0.29 (0.01–2.55)	0.38 ± 0.42	0.27 (0.01–1.42)	0.614	0.959
	**Bulgur** **(26/5)**	0.36 ± 0.32	0.27 (0.07–1.45)	0.74 ± 0.59	0.45 (0.10–1.46)	0.45 ± 0.35	0.36 (0.04–1.31)	0.44 ± 0.47	0.22 (0.02–1.01)	0.192	0.080
	**Quinoa** **(26/5)**	0.49 ± 0.48	0.28 (0.021–1.53)	0.42 ± 0.29	0.29 (0.16–0.78)	0.33 ± 0.19	0.31 (0.17–0.71)	0.63 ± 0.72	0.32 (0.19–1.92)	0.174	0.345
	**Buckwheat (26/6)**	0.35 ± 0.25	0.29 (0.09–1.12)	0.25 ± 0.21	0.18 (0.01–0.55)	0.37 ± 0.28	0.28 (0.08–1.05)	0.16 ± 0.15	0.16 (0.01–0.33)	0.603	0.082
***p*** **^between-groups^**		0.956		0.369		0.724		0.960			
**TNF-α** **(pg/mL)**	**Rice** **(21/10)**	11.07 ± 14.85	5.09 (0.17–61.30)	5.77 ± 5.19	4.94 (0.00–15.26)	9.98 ± 10.43	4.92 (0.02–33.32)	5.52 ± 6.45	4.06 (0.07–22.07)	0.795	0.799
	**Bulgur** **(26/5)**	10.40 ± 15.73	7.37 (0.78–80.02)	7.26 ± 6.42	9.76 (0.06–13.69)	12.09 ± 17.95	6.72 (0.06–85.90)	5.99 ± 9.24	2.24 (0.64–22.10)	0.316	0.686
	**Quinoa** **(26/5)**	11.42 ± 19.18	4.50 (0.92–80.02)	2.08 ± 1.58	2.39 (0.07–4.15)	8.88 ± 10.35	4.96 (0.08–35.47)	2.01 ± 1.48	2.27 (0.89–4.04)	0.545	0.498
	**Buckwheat (26/6)**	13.21 ± 15.34	7.08 (0.32–65.57)	9.93 ± 17.84	3.15 (0.38–46.07)	8.69 ± 8.55	5.75 (0.24–34.44)	2.59 ± 2.27	2.29 (0.21–6.62)	0.143	0.463
***p*** **^between-groups^**		0.534		0.665		0.948		0.669			
**SBP** **(mm-Hg)**	**Rice** **(21/10)**	117.86 ± 7.77	120.00 (100.00–130.00)	116.00 ± 10.49	120.00 (100.00–130.00)	120.67 ± 8.56	120.00 (105.00–130.00)	120.70 ± 12.84	120.00 (95.00–140.00)	0.092	0.079
	**Bulgur** **(26/5)**	117.00 ± 10.73	119.00 (100.00–140.00)	122.40 ± 10.31	120.00 (115.00–140.00)	117.96 ± 10.88	120.00 (90.00–130.00)	122.60 ± 25.47	118.00 (90.00–160.00)	0.486	0.715
	**Quinoa** **(26/5)**	114.96 ± 15.44	120.00 (90.00–135.00)	125.00 ± 8.66	130.00 (110.00–130.00)	115.54 ± 8.37	117.00 (100.00–125.00)	117.00 ± 6.71	120.00 (110.00–125.00)	0.951	0.064
	**Buckwheat (26/6)**	118.85 ± 13.48	118.50 (85.00–143.00)	120.00 ± 8.37	120.00 (110.00–135.00)	113.92 ± 9.81	113.50 (90.00–135.00)	112.00 ± 9.90	110.00 (100.00–127.00)	0.018 *	0.066
***p*** **^between-groups^**		0.913		0.384		0.064		0.423			
**DBP** **(mm-Hg)**	**Rice** **(21/10)**	74.81 ± 7.85	75.00 (60.00–90.00)	76.50 ± 7.09	80.00 (65.00–85.00)	77.76 ± 9.41	75.00 (65.00–100.00)	78.20 ± 6.43	80.00 (65.00–85.00)	0.166	0.257
	**Bulgur** **(26/5)**	74.27 ± 8.09	72.50 (60.00–100.00)	76.00 ± 8.22	75.00 (70.00–90.00)	76.85 ± 7.32	75.00 (70.00–100.00)	73.00 ± 10.95	70.00 (60.00–90.00)	0.196	0.180
	**Quinoa** **(26/5)**	72.08 ± 8.89	72.50 (60.00–86.00)	77.00 ± 7.58	80.00 (65.00–85.00)	72.35 ± 6.01	70.00 (60.00–80.00)	76.00 ± 2.24	75.00 (75.00–80.00)	0.910	0.785
	**Buckwheat (26/6)**	75.69 ± 7.99	75.00 (55.00–90.00)	78.33 ± 5.16	80.00 (70.00–85.00)	73.35 ± 5.58	70.00 (60.00–85.00)	70.00 ± 5.48	70.00 (60.00–75.00)	0.042 *	0.059
***p*** **^between-groups^**		0.489		0.891		0.072		0.074			

F: Female, M: Male. Data were represented as mean ± standard deviation and median (minimum–maximum). Statistically significant *p* values (*p* < 0.05) were indicated as bold and with an asterisk (*). p between-groups values were obtained from Kruskal–Wallis test. *p* within (F) and *p* within (M) represent *p* values obtained by Wilcoxon test in female and male participants within each group, respectively.

**Table 4:** Values of incremental area under curve (iAUC) for glucose and insulin levels.

		Pre-Intervention				Post-Intervention					
		Female (*n* = 99)		Male (*n* = 26)		Female (*n* = 99)		Male (*n* = 26)		*p* ^within^	
	Groups (F/M)	X ± SD	Median (Min–Max)	X ± SD	Median (Min–Max)	X ± SD	Median (Min–Max)	X ± SD	Median (Min–Max)	*p* ^within (F)^	*p* ^within (M)^
**iAUC** **Glucose**	**Rice** **(21/10)**	4353.66 ± 2110.12	4545.00 (330.00–7845.00)	4550.25 ± 2365.48	3877.50 **^ab^** (2119.18–10,215.00)	4208.41 ± 2067.23	4012.57 (1168.06–7875.00)	4651.22 ± 3789.39	4271.70 (581.54–14,415.00)	0.575	0.959
	**Bulgur** **(26/5)**	4029.34 ± 3025.22	3547.50 (0.00–11,460.00)	1930.40 ± 728.09	1722.22 **^b^** (1275.34–3180.00)	4333.47 ± 3058.64	3167.50 (544.66–14,205.00)	3595.73 ± 1356.77	3078.64 (2157.69–5520.00)	0.151	0.043 *
	**Quinoa** **(26/5)**	3693.85 ± 2365.16	3300.00 (131.54–8385.00)	5320.03 ± 2663.36	4710.00 **^a^** (2566.73–9510.00)	3132.64 ± 2428.18	2743.39 (0.00–11,370.00)	5977.55 ± 2573.41	6540.00 (1537.73–8070.00)	0.200	0.500
	**Buckwheat (26/6)**	3416.94 ± 2357.28	3052.50 (4.77–8400.00)	3551.41 ± 2093.67	2824.50 **^ab^** (1976.36–7485.00)	2825.73 ± 1957.17	2818.43 (113.44–6840.00)	4361.10 ± 1718.91	4387.50 (2106.60–7065.00)	0.269	0.075
***p*** **^between-groups^**		0.495		0.040 *		0.900		0.301			
**iAUC** **Insulin**	**Rice** **(21/10)**	5531.54 ± 3940.22	5127.75 (1174.50–15,454.34)	14,131.33 ± 20,436.89	8368.95 (4646.25–71,736.00)	5230.76 ± 3169.49	4422.30 (1911.45–14,029.05)	15,115.24 ± 28,093.00	5785.16 (469.18–94,372.20)	0.876	0.333
	**Bulgur** **(26/5)**	5124.77 ± 3552.73	3347.47 (1647.15–13,311.15)	6978.81 ±1633.84	7215.00 (4791.75–8644.50)	5396.02 ± 4571.37	4144.50 (1298.85–22,292.70)	6257.10 ±1152.70	5482.55 (5376.90–7735.85)	0.790	0.345
	**Quinoa** **(26/5)**	5489.16 ± 3938.49	4886.93 (1077.94–20,978.70)	5748.21 ± 2206.83	5685.00 (3273.45–8729.70)	5287.64 ± 2780.48	4956.00 (1188.45–13,681.50)	6141.23 ± 2368.91	6423.00 (2600.19–8298.00)	0.949	0.225
	**Buckwheat (26/6)**	6144.74 ± 4040.34	4046.78 (1693.74–15,457.05)	7443.82 ± 6513.50	5058.00 (1060.03–18,989.10)	5573.02 ± 3650.89	4923.75 (976.05–14,454.60)	6030.25 ± 6340.89	3719.10 (1301.85–18,131.85)	0.354	0.046 *
***p*** **^between-groups^**		0.802		0.403		0.888		0.307			

F: Female, M: Male. Values were represented as mean ± standard deviation and median (minimum–maximum). Statistically significant *p* values (*p* < 0.05) were indicated as bold and with an asterisk (*). p between-group values were obtained by Kruskal–Wallis test. p within (F) and p within (M) represent *p* values obtained by the Wilcoxon test in female and male participants within each group, respectively. Different superscript letters (a–b) within the same column indicate a statistically significant difference between the groups based on Dunn’s post hoc pairwise comparisons following the Kruskal–Wallis test (*p* < 0.05).

**Table 5:** Anthropometrical measurements of groups before and after the intervention.

		Pre-Intervention				Post-Intervention					
	Female (*n* = 99)			Male (*n* = 26)		Female (*n* = 99)		Male (*n* = 26)			
	Groups (F/M)	X ± SD	Median (Min–Max)	X ± SD	Median (Min–Max)	X ± SD	Median (Min–Max)	X ± SD	Median (Min–Max)	*p* ^within (F)^	*p* ^within^ ^(M)^
**Body** **Weight (kg)**	**Rice** **(21/10)**	78.53 ± 11.30	76.20 (64.80–106.90)	96.43 ± 10.94	95.80 (81.40–119.00)	77.49 ± 11.22	75.10 (62.80–106.00)	96.08 ± 10.91	95.10 (83.10–119.60)	0.002 *	0.003 *
	**Bulgur** **(26/5)**	82.43 ± 11.88	81.05 (66.60–110.70)	96.96 ± 9.99	99.60 (80.10–106.50)	81.55 ± 11.66	80.75 (65.50–110.40)	96.52 ± 10.45	98.00 (79.50–107.70)	0.010 *	0.006 *
	**Quinoa** **(26/5)**	79.69 ± 8.99	78.40 (66.80–99.80)	85.66 ± 5.81	83.20 (80.10–92.20)	79.38 ± 9.10	77.90 (65.20–101.50)	85.52 ± 5.29	83.00 (81.00–92.20)	0.395	0.416
	**Buckwheat** **(26/6)**	79.16 ± 12.07	76.70 (60.40–101.70)	87.52 ± 11.77	83.70 (73.60–106.50)	77.83 ± 11.75	75.65 (59.20–99.80)	85.73 ± 10.78	81.95 (72.80–102.90)	0.002 *	<0.001*
***p*** **^between-groups^**		0.620		0.140		0.539		0.076			
**BMI** **(kg/m^2^)**	**Rice** **(21/10)**	30.22 ± 3.22	30.40 (25.30–34.90)	31.13 ± 2.48	31.25 (26.00–34.50)	29.82 ± 3.10	30.00 (24.50–35.20)	30.91 ± 2.30	31.10 (26.50–34.40)	0.002 *	0.001 *
	**Bulgur** **(26/5)**	31.13 ± 3.32	32.15 (25.32–35.20)	31.58 ± 3.04	32.60 (26.80–34.40)	30.89 ± 3.34	31.55 (24.90–35.70)	31.38 ± 3.30	32.20 (26.30–34.80)	0.044 *	0.019 *
	**Quinoa** **(26/5)**	29.52 ± 2.89	29.25 (25.20–34.90)	28.88 ± 1.77	29.20 (26.00–30.80)	29.40 ± 2.96	28.70 (24.20–35.60)	28.72 ± 1.56	29.10 (26.00–29.90)	0.389	0.367
	**Buckwheat** **(26/6)**	29.66 ± 3.58	28.60 (25.00–35.00)	29.40 ± 2.72	28.55 (26.70–32.90)	29.20 ± 3.58	28.20 (23.40–35.90)	28.78 ± 2.41	27.85 (26.10–32.10)	0.005 *	0.001 *
***p*** **^between-groups^**		0.305		0.206		0.244		0.095			
**Waist/Hip** **Ratio**	**Rice** **(21/10)**	0.86 ± 0.06	0.86 (0.76–0.98)	0.97 ± 0.06	0.96 (0.89–1.09)	0.86 ± 0.07	0.86 (0.75–1.06)	0.97 ± 0.06	0.96 (0.87–1.04)	0.575	0.495
	**Bulgur** **(26/5)**	0.86 ± 0.05	0.86 (0.75–0.96)	0.97 ± 0.09	0.98 (0.86–1.10)	0.85 ± 0.06	0.85 (0.74–0.96)	0.97 ± 0.09	0.97 (0.87–1.08)	0.530	0.484
	**Quinoa** **(26/5)**	0.85 ± 0.06	0.83 (0.68–1.00)	0.95 ± 0.04	0.96 (0.91–1.01)	0.84 ± 0.05	0.83 (0.70–0.93)	0.96 ± 0.04	0.95 (0.90–1.02)	0.373	0.527
	**Buckwheat** **(26/6)**	0.85 ± 0.08	0.85 (0.72–1.04)	0.95 ± 0.09	0.96 (0.84–1.04)	0.84 ± 0.08	0.84 (0.70–1.02)	0.94 ± 0.07	0.94 (0.87–1.03)	0.177	0.249
***p*** **^between-groups^**		0.814		0.941		0.747		0.834			
**Fat Mass (%)**	**Rice** **(21/10)**	35.80 ± 3.63	36.20 (30.30–42.00)	27.34 ± 3.28	27.30 (19.90–31.90)	35.90 ± 3.93	36.70 (29.30–43.10)	26.30 ± 3.52	26.45 (17.90–31.20)	0.794	0.221
	**Bulgur** **(26/5)**	35.59 ± 4.67	35.00 (27.70–44.30)	27.34 ± 4.43	28.50 (20.20–31.60)	35.91 ± 4.41	36.25 (26.80–43.60)	27.10 ± 5.22	29.10 (18.70–32.00)	0.753	0.922
	**Quinoa** **(26/5)**	35.58 ± 3.31	36.20 (30.70–41.50)	25.08 ± 3.11	26.20 (21.10–28.00)	35.07 ± 3.48	35.10 (29.20–42.50)	23.52 ± 3.89	24.70 (17.40–27.00)	0.022 *	0.004 *
	**Buckwheat** **(26/6)**	35.33 ± 3.65	35.15 (28.90–43.40)	24.63 ± 3.18	24.05 (20.20–28.90)	34.74 ± 3.74	34.95 (27.30–43.40)	23.62 ± 3.59	22.95 (19.00–28.30)	0.027 *	0.006 *
***p*** **^between-groups^**		0.959		0.401		0.586		0.213			
**Fat-free Mass (kg)**	**Rice** **(21/10)**	50.01 ± 5.74	48.40 (42.10–67.40)	69.87 ± 5.62	69.70 (63.00–82.00)	49.52 ± 5.90	47.60 (42.50–66.70)	70.68 ± 6.37	70.15 (62.70–85.20)	0.161	0.902
	**Bulgur** **(26/5)**	52.88 ± 6.95	50.35 (44.20–71.80)	69.84 ± 3.86	70.90 (63.90–73.30)	51.95 ± 5.88	50.20 (44.00–66.90)	69.92 ± 3.56	70.70 (64.60–73.20)	0.091	0.115
	**Quinoa** **(26/5)**	51.25 ± 4.57	50.80 (44.80–66.70)	64.48 ± 6.02	65.60 (58.00–71.00)	51.37 ± 4.94	50.95 (46.00–68.00)	65.42 ± 5.64	67.90 (59.10–71.70)	0.477	0.235
	**Buckwheat** **(26/6)**	50.99 ± 6.82	50.15 (40.80–68.20)	65.53 ± 6.56	65.45 (56.60–75.70)	50.40 ± 6.01	49.05 (40.30–63.50)	65.22 ± 6.04	64.90 (57.10–75.10)	0.137	0.097
***p*** **^between-groups^**			0.511	0.325		0.315		0.211			

F: Female, M: Male. Values were represented as mean ± standard deviation and median (minimum–maximum). Statistically significant *p* values (*p* < 0.05) were indicated as bold and with an asterisk (*). p ^between-groups^ values were obtained by Kruskal–Wallis test. p ^within (F)^ and p ^within (M)^ represent *p* values obtained by the Wilcoxon test in female and male participants within each group, respectively.

**Table 6:** Energy and macronutrients intakes of groups in pre-intervention period and during intervention period.

	Pre-Intervention					During-Intervention					
		Female (*n* = 99)		Male (*n* = 26)		Female (*n* = 99)		Male (*n* = 26)			
	Groups (F/M)	X ± SD	Median (Min–Max)	X ± SD	Median (Min–Max)	X ± SD	Median (Min–Max)	X ± SD	Median (Min–Max)	*p* ^within^ ^(F)^	*p* ^within (M)^
**Energy** **(kcal)**	**Rice** **(21/10)**	1675.45 ± 342.30	1665.34 (877.56–2228.50)	2416.36 ± 522.48	2332.23 (1626.73–3034.85)	1389.91 ± 299.89	1371.34 (686.38–1997.11)	2077.22 ± 592.79	2151.73 (1204.22–2856.70)	0.002 *	0.037 *
	**Bulgur** **(26/5)**	1618.06 ± 689.97	1506.32 (769.24–3586.23)	2231.62 ± 877.91	1993.83 (1416.53–3487.66)	1557.30 ± 504.33	1498.44 (808.85–3002.17)	1749.23 ± 444.01	1763.08 (1229.54–2214.77)	0.675	0.043 *
	**Quinoa** **(26/5)**	1531.90 ± 452.69	1507.07 (803.72–2371.00)	2571.50 ± 475.52	2730.81 (2039.39–3124.76)	1555.09 ± 558.51	1521.41 (758.82–3200.74)	2262.32 ± 417.94	2110.73 (1915.28–2960.09)	0.949	0.045 *
	**Buckwheat** **(26/6)**	1876.68 ± 521.51	1831.32 (1008.63–2988.84)	2067.33 ± 492.82	2010.85 (1404.09–2668.07)	1637.47 ± 461.24	1657.81 (1024.94–2800.75)	2072.41 ± 595.83	2087.83 (1246.87–2867.32)	0.019 *	0.917
***p*** **^between-groups^**		0.058		0.360			0.357	0.533			
**Carbohyd-** **rate (g)**	**Rice** **(21/10)**	43.68 ± 5.34	43.80 (32.40–55.40)	43.34 ± 6.01	44.30 (32.40–51.20)	46.66 ± 7.57	46.25 **^a^** (32.25–59.50)	47.20 ± 3.74	46.50 (40.50–52.00)	0.052	0.028 *
	**Bulgur** **(26/5)**	45.73 ± 25.36	41.80 (29.00–166.81)	42.40 ± 4.45	44.60 (35.60–46.20)	41.15 ± 5.91	41.62 **^ab^** (30.75–49.50)	44.95 ± 9.34	43.25 (34.25–58.50)	0.929	0.498
	**Quinoa** **(26/5)**	42.06 ± 7.51	43.00 (21.20–55.20)	44.76 ± 5.31	47.00 (38.40–51.20)	42.88 ± 6.21	43.00 **^ab^** (31.00–57.25)	44.95 ± 7.23	44.50 (37.50–56.00)	0.778	0.988
	**Buckwheat** **(26/6)**	41.32 ± 4.35	41.67 (29.00–48.60)	38.23 ± 6.55	40.70 (25.80–43.60)	40.82 ± 4.27	39.75 **^b^** (32.50–51.25)	41.62 ± 12.09	43.00 (22.50–54.00)	0.402	0.345
***p*** **^between-groups^**		0.421		0.356		0.008 *		0.777			
**Protein (g)**	**Rice** **(21/10)**	14.16 ± 2.39	14.00 (9.40–18.00)	15.88 ± 2.61	16.60 (11.40–20.40)	14.39 ± 2.74	13.75 **^a^** (10.50–19.75)	16.70 ± 2.51	17.12 (12.00–19.50)	0.751	0.241
	**Bulgur** **(26/5)**	17.22 ± 8.13	15.50 (12.00–53.80)	13.76 ± 1.13	13.60 (12.20–15.20)	16.39 ± 3.01	16.25 **^ab^** (9.00–23.00)	16.45 ± 2.17	15.50 (14.75–20.00)	0.509	0.043 *
	**Quinoa** **(26/5)**	15.01 ± 2.34	14.50 (10.80–20.00)	14.84 ± 2.41	14.60 (12.20–18.40)	16.60 ± 2.44	16.62 **^b^** (11.75–21.25)	16.25 ± 1.35	16.00 (14.25–17.50)	0.006 *	0.225
	**Buckwheat** **(26/6)**	15.31 ± 2.75	15.00 (10.20–20.60)	17.53 ± 3.23	17.00 (13.80–22.60)	16.36 ± 2.57	16.00 **^ab^** (12.00–24.50)	18.54 ± 4.89	16.75 (13.25–25.00)	0.134	0.600
***p*** **^between-groups^**		0.342		0.105		0.020 *		0.930			
**Fat (g)**	**Rice** **(21/10)**	42.14 ± 4.79	42.60 (33.00–53.20)	40.34 ± 4.40	39.90 (33.80–47.20)	38.97 ± 7.26	37.50 (29.25–53.50)	36.15 ± 2.82	36.38 (31.25–40.00)	0.052	0.022 *
	**Bulgur** **(26/5)**	45.05 ± 10.29	43.60 (31.80–88.26)	43.76 ± 4.12	41.20 (40.40–49.20)	42.44 ± 4.85	42.12 (33.75–52.25)	38.55 ± 8.27	40.25 (26.25–48.75)	0.275	0.043 *
	**Quinoa** **(26/5)**	42.35 ± 6.17	42.00 (31.80–58.40)	40.36 ± 3.95	39.60 (36.20–45.40)	40.44 ± 5.11	40.62 (28.50–51.25)	38.50 ± 7.97	38.50 (26.50–47.00)	0.141	0.500
	**Buckwheat** **(26/6)**	43.23 ± 4.24	43.30 (34.40–52.80)	43.47 ± 3.44	44.40 (37.20–47.40)	42.47 ± 4.17	42.88 (34.00–49.25)	37.62 ± 5.70	39.62 (29.50–44.00)	0.485	0.028 *
***p*** **^between-groups^**		0.685	0.332				0.071	0.592			

F: Female, M: Male. Values were represented as mean ± standard deviation and median (minimum–maximum). Statistically significant *p* values (*p* < 0.05) were indicated as bold and with an asterisk (*). p ^between-groups^ values were obtained by Kruskal–Wallis test. p ^within (F)^ and p ^within (M)^ represent *p* values obtained from Wilcoxon test in female and male participants within each group, respectively. Different superscript letters (a–b) within the same column indicate a statistically significant difference between groups based on Dunn’s post hoc pairwise comparisons following the Kruskal–Wallis test (*p* < 0.05).

## Data Availability

The data presented in this study are available on request from the corresponding author due to ethical and privacy restrictions related to participant health information collected during the clinical trial.

## Supplementary Materials

The following supporting information can be downloaded at https://www.mdpi.com/article/10.3390/metabo16090628/s1, Table S1: CONSORT guideline checklist.

## Abbreviations

The following abbreviations are used in this manuscript:

**List of Abbreviations (Alphabetical Order)**	
ACE	Angiotensin Converting Enzyme
ALT	Alanine Aminotransferase
AST	Aspartate Aminotransferase
DBP	Diastolic Blood Pressure
BMI	Body Mass Index
CONSORT	Consolidated Standards for Reporting Trials
ELISA	Enzyme Linked Immunosorbent Analysis
GGT	Gamma-Glutamyl Transferase
HbA1c	Hemoglobin A1c
HDL-C	High-Density Lipoprotein Cholesterol
HOMA-IR	Homeostasis Model Assessment of Insulin Resistance
iAUC	Incremental Area Under Curve
LDL-C	Low-Density Lipoprotein Cholesterol
MDA	Malondialdehyde
OGTT	Oral Glucose Tolerance Test
SBP	Systolic Blood Pressure
SCFA	Short Chain Fatty Acid
T2DM	Type 2 Diabetes Mellitus
TC	Total Cholesterol
TG	Triglycerides

## References

[1] Hardy D.S., Garvin J.T., Xu H. (2020). Carbohydrate Quality, Glycemic Index, Glycemic Load and Cardiometabolic Risks in the US, Europe and Asia: A Dose e Response Meta-Analysis. Nutr. Metab. Cardiovasc. Dis..

[2] Kazeminasab F., Miraghajani M., Khalafi M., Sakhaei M.H., Rosenkranz S.K., Santos H.O. (2024). Effects of Low-Carbohydrate Diets, with and without Caloric Restriction, on Inflammatory Markers in Adults: A Systematic Review and Meta-Analysis of Randomized Clinical Trials. Eur. J. Clin. Nutr..

[3] Food and Agriculture Organization (FAO) Cereal Supply and Demand Brief. http://www.fao.org/worldfoodsituation/csdb/en/.

[4] Carcea M. (2021). Value of Wholegrain Rice in a Healthy Human Nutrition. Agriculture.

[5] Tagliapietra B.L., Flores Soares C., Pedrosa M.T., Clerici S. (2024). Rice (*Oryza sativa* L.) and Its Products for Human Consumption: General Characteristics, Nutritional Properties, and Types of Processing. Food Sci. Technol..

[6] Stone A.K., Wang S., Tulbek M., Koksel F., Nickerson M.T. (2020). Processing and Quality Aspects of Bulgur from *Triticum durum*. Cereal Chem..

[7] United States Department of Agriculture Bulgur, (Dry, Raw). FoodData Central. https://fdc.nal.usda.gov/food-details/2710820/nutrients.

[8] Shah Y.A., Saeed F., Afzaal M., Ahmad A., Hussain M., Ateeq H., Khan M.H. (2022). Biochemical and Nutritional Properties of Wheat Bulgur: A Review. J. Food Process. Preserv..

[9] Kaplan Evlice A., Özkaya H. (2020). Effects of Wheat Cultivar, Cooking Method, and Bulgur Type on Nutritional Quality Characteristics of Bulgur. J. Cereal Sci..

[10] Pérez-Viveros D.J., Vargas-Torres A., Gómez-Aldapa C.A., Cruz-Cansino N.d.S., Hernández-Uribe J.P. (2026). Comparative Analysis of White, Red, and Black Quinoa: Nutritional Composition, Technological Functionality, and Health Implications. J. Food Sci..

[11] Brito I.d.L., Chantelle L., Magnani M., Cordeiro A.M.T.d.M. (2022). Nutritional, Therapeutic, and Technological Perspectives of Quinoa (*Chenopodium quinoa* Willd.): A Review. J. Food Process. Preserv..

[12] Lițoiu A.A., Păucean A., Lung C., Zmuncilă A., Chiș M.S. (2025). An Overview of Buckwheat—A Superfood with Applicability in Human Health and Food Packaging. Plants.

[13] Zamaratskaia G., Gerhardt K., Knicky M., Wendin K. (2024). Buckwheat: An Underutilized Crop with Attractive Sensory Qualities and Health Benefits. Crit. Rev. Food Sci. Nutr..

[14] Sonawane S., Shams R., Dash K.K., Patil V., Pandey V.K., Dar A.H. (2024). Nutritional Profile, Bioactive Properties and Potential Health Benefits of Buckwheat: A Review. eFood.

[15] Gao J., Kreft I., Chao G., Wang Y., Liu X., Wang L., Wang P., Gao X., Feng B. (2016). Tartary Buckwheat (*Fagopyrum tataricum* Gaertn.) Starch, a Side Product in Functional Food Production, as a Potential Source of Retrograded Starch. Food Chem..

[16] Gorinstein S., Lojek A., Číž M., Pawelzik E., Delgado-Licon E., Medina O.J., Moreno M., Salas I.A., Goshev I. (2008). Comparison of Composition and Antioxidant Capacity of Some Cereals and Pseudocereals. Int. J. Food Sci. Technol..

[17] Zhu F. (2019). Proanthocyanidins in Cereals and Pseudocereals. Crit. Rev. Food Sci. Nutr..

[18] Nandan A., Koirala P., Dutt Tripathi A., Vikranta U., Shah K., Gupta A.J., Agarwal A., Nirmal N. (2024). Nutritional and Functional Perspectives of Pseudocereals. Food Chem..

[19] Mir N.A., Riar C.S., Singh S. (2018). Nutritional Constituents of Pseudo Cereals and Their Potential Use in Food Systems: A Review. Trends Food Sci. Technol..

[20] Kaur H., Shams R., Dash K.K., Dar A.H. (2023). A Comprehensive Reiew of Pseudo-Cereals: Nutritional Profile, Phytochemicals Constituents and Potential Health Promoting Benefits. Appl. Food Res..

[21] Wang Q., Yao L., Li Q., Xie H., Guo Y., Huang T., Zhang X., Liu J., Zhang P., Li L. (2022). Integrative Analysis of the Metabolome and Transcriptome Provides Insights into the Mechanisms of Flavonoid Biosynthesis in Quinoa Seeds at Different Developmental Stages. Metabolites.

[22] Oztekin Y., Buyuktuncer Z. (2026). A Narrative Review on Pseudocereals and Cardiometabolic Health: Biological Mechanisms and Evidence from Human Studies. Nutrients.

[23] Garcia Tejedor A., Haros C.M., Laparra Llopis J.M. (2023). Chenopodium Quinoa’s Ingredients Improve Control of the Hepatic Lipid Disturbances Derived from a High-Fat Diet. Foods.

[24] Wu W., Wang L., Qiu J., Li Z. (2018). The Analysis of Fagopyritols from Tartary Buckwheat and Their Anti-Diabetic e Ff Ects in KK-A y Type 2 Diabetic Mice and HepG2 Cells. J. Funct. Foods.

[25] Foucault A.S., Mathé V., Lafont R., Even P., Dioh W., Veillet S., Tomé D., Huneau J.F., Hermier D., Quignard-Boulangé A. (2012). Quinoa Extract Enriched in 20-Hydroxyecdysone Protects Mice from Diet-Induced Obesity and Modulates Adipokines Expression. Obesity.

[26] Pourshahidi L.K., Caballero E., Osses A., Hyland B.W., Ternan N.G., Gill C.I.R. (2020). Modest Improvement in CVD Risk Markers in Older Adults Following Quinoa (*Chenopodium quinoa* Willd.) Consumption: A Randomized-Controlled Crossover Study with a Novel Food Product. Eur. J. Nutr..

[27] Farinazzi-Machado F.M.V., Barbalho S.M., Oshiiwa M., Goulart R., Pessan Junior O. (2012). Use of Cereal Bars with Quinoa (*Chenopodium quinoa* W.) to Reduce Risk Factors Related to Cardiovascular Diseases. Food Sci. Technol..

[28] Navarro-Perez D., Radcliffe J., Tierney A., Jois M. (2017). Quinoa Seed Lowers Serum Triglycerides in Overweight and Obese Subjects: A Dose- Response Randomized Controlled Clinical Trial. Curr. Dev. Nutr..

[29] Stokić E., Mandić A., Sakač M., Mišan A., Pestorić M., Šimurina O., Jambrec D., Jovanov P., Nedeljković N., Milovanović I. (2015). Quality of Buckwheat-Enriched Wheat Bread and Its Antihyperlipidemic Effect in Statin Treated Patients. LWT.

[30] Wieslander G., Fabjan N., Vogrincic M., Kreft I., Janson C., Spetz-Nyström U., Vombergar B., Tagesson C., Leanderson P., Norbäck D. (2011). Eating Buckwheat Cookies Is Associated with the Reduction in Serum Levels of Myeloperoxidase and Cholesterol: A Double Blind Crossover Study in Day-Care Centre Staffs. Tohoku J. Exp. Med..

[31] Mišan A., Petelin A., Stubelj M., Mandić A., Šimurina O., Pojić M., Milovanović I., Jakus T., Filipčev B., Jenko Pražnikar Z. (2017). Buckwheat—Enriched Instant Porridge Improves Lipid Profile and Reduces Inflammation in Participants with Mild to Moderate Hypercholesterolemia. J. Funct. Foods.

[32] Qiu J., Liu Y., Yue Y., Qin Y., Li Z. (2016). Dietary Tartary Buckwheat Intake Attenuates Insulin Resistance and Improves Lipid Profiles in Patients with Type 2 Diabetes: A Randomized Controlled Trial. Nutr. Res..

[33] De Carvalho F.G., Ovídio P.P., Padovan G.J., Jordão Junior A.A., Marchini J.S., Navarro A.M. (2014). Metabolic Parameters of Postmenopausal Women after Quinoa or Corn Flakes Intake—A Prospective and Double-Blind Study. Int. J. Food Sci. Nutr..

[34] Nishimura M., Ohkawara T., Sato Y., Satoh H., Suzuki T., Ishiguro K., Noda T., Morishita T., Nishihira J. (2016). Effectiveness of Rutin-Rich Tartary Buckwheat (*Fagopyrum tataricum* Gaertn.) ‘Manten-Kirari’ in Body Weight Reduction Related to Its Antioxidant Properties: A Randomised, Double-Blind, Placebo-Controlled Study. J. Funct. Foods.

[35] Johnston C.S., Snyder D., Smith C. (2017). Commercially Available Gluten-Free Pastas Elevate Postprandial Glycemia in Comparison to Conventional Wheat Pasta in Healthy Adults: A Double-Blind Randomized Crossover Trial. Food Funct..

[36] Gabrial S.G.N., Shakib M.C.R., Gabrial G.N. (2016). Effect of Pseudocereal-Based Breakfast Meals on the First and Second Meal Glucose Tolerance in Healthy and Diabetic Subjects. Open Access Maced. J. Med. Sci..

[37] Stringer D.M., Taylor C.G., Appah P., Blewett H., Zahradka P. (2013). Consumption of Buckwheat Modulates the Post-Prandial Response of Selected Gastrointestinal Satiety Hormones in Individuals with Type 2 Diabetes Mellitus. Metabolism.

[38] Vetrani C., Bozzetto L., Giorgini M., Cavagnuolo L., Di Mattia E., Cipriano P., Mangione A., Todisco A., Inghilterra G., Giacco A. (2019). Fibre-Enriched Buckwheat Pasta Modifies Blood Glucose Response Compared to Corn Pasta in Individuals with Type 1 Diabetes and Celiac Disease: Acute Randomized Controlled Trial. Diabetes Res. Clin. Pract..

[39] Díaz-Rizzolo D.A., Acar-Denizli N., Kostov B., Roura E., Sisó-Almirall A., Delicado P., Gomis R. (2022). Glycaemia Fluctuations Improvement in Old-Age Prediabetic Subjects Consuming a Quinoa-Based Diet: A Pilot Study. Nutrients.

[40] Li L., Lietz G., Bal W., Watson A., Morfey B., Seal C. (2018). Effects of Quinoa (*Chenopodium quinoa* Willd.) Consumption on Markers of CVD Risk. Nutrients.

[41] Defries D.M., Petkau J.C., Gregor T., Blewett H. (2018). A Randomized, Controlled, Crossover Study of Appetite-Related Sensations after Consuming Snacks Made from Buckwheat. Appl. Physiol. Nutr. Metab..

[42] Hopewell S., Chan A.W., Collins G.S., Hróbjartsson A., Moher D., Schulz K.F., Tunn R., Aggarwal R., Berkwits M., Berlin J.A. (2025). CONSORT 2025 Statement: Updated Guideline for Reporting Randomised Trials. BMJ.

[43] NCSS (2024). PASS 2024 Power Analysis and Sample Size Software 2024.

[44] Llanaj E., Ahanchi N.S., Dizdari H., Taneri P.E., Niehot C.D., Wehrli F., Khatami F., Raeisi-Dehkordi H., Kastrati L., Bano A. (2022). Buckwheat and Cardiometabolic Health: A Systematic Review and Meta-Analysis. J. Pers. Med..

[45] Zeng H., Cai X., Qiu Z., Liang Y., Huang L. (2023). Glucolipid Metabolism Improvement in Impaired Glucose Tolerance Subjects Consuming a Quinoa-Based Diet: A Randomized Parallel Clinical Trial. Front. Physiol..

[46] Huang H., Jia C., Chen X., Zhang L., Jiang Y., Meng X., Liu X. (2024). Progress in Research on the Effects of Quinoa (*Chenopodium quinoa*) Bioactive Compounds and Products on Intestinal Flora. Front. Nutr..

[47] USDA FoodData Central. https://fdc.nal.usda.gov/.

[48] Thakur P., Kumar K., Singh H. (2021). Food Bioscience Nutritional Facts, Bio-Active Components and Processing Aspects of Pseudocereals: A Comprehensive Review. Food Biosci..

[49] Devi W.D., Morya S., Bashir O., Kara M., Mugabi R. (2026). Comprehensive Review on Functional Foods from Pseudo-Cereals: Insight to Nutritional Properties and Processing Techniques. Discov. Appl. Sci..

[50] Sekarmuti A., Rimbawan R., Nasution Z. (2025). Effects of Cooking Techniques on the Nutritional Profile, Glycemic Index, and Sensory Evaluation of Rice: A Systematic Review. Prev. Nutr. Food Sci..

[51] Wellens J., Vissers E., Dumoulin A., Hoekx S., Vanderstappen J., Verbeke J., Vangoitsenhoven R., Derrien M., Verstockt B., Ferrante M. (2025). Cooking Methods Affect Advanced Glycation End Products and Lipid Profiles: A Randomized Cross-over Study in Healthy Subjects. Cell Rep. Med..

[52] Huang L., Li X., Zou M., Zeng H., Wu S., Liang Y., Wang D., Yang Y., Qiu Z., Zhou Q. (2025). Quinoa Is More Effective than Other Whole Grains in the Management of Impaired Glucose Tolerance: A Randomized Controlled Trial. Food Funct..

[53] Colasanto A., Savastio S., Pozzi E., Gorla C., Coïsson J.D., Arlorio M., Rabbone I. (2023). The Impact of Different Types of Rice and Cooking on Postprandial Glycemic Trends in Children with Type 1 Diabetes with or without Celiac Disease. Nutrients.

[54] Buckley J.D., Thorp A.A., Murphy K.J., Howe P.R.C. (2006). Dose-Dependent Inhibition of the Post-Prandial Glycaemic Response to a Standard Carbohydrate Meal Following Incorporation of Alpha-Cyclodextrin. Ann. Nutr. Metab..

[55] Kameyama N., Maruyama C., Matsui S., Araki R., Yamada Y., Maruyama T. (2014). Effects of Consumption of Main and Side Dishes with White Rice on Postprandial Glucose, Insulin, Glucose-Dependent Insulinotropic Polypeptide and Glucagon-like Peptide-1 Responses in Healthy Japanese Men. Br. J. Nutr..

[56] World Health Organization Waist Circumference and Waist-Hip Ratio: Report of a WHO Expert Consultation. https://www.who.int/publications/i/item/9789241501491.

[57] Wolever T.M.S., Jenkins D.J.A., Jenkins A.L., Josse R.G. (1991). The Glycemic Index: Methodology and Clinical Implications. Am. J. Clin. Nutr..

[58] Foucault A.S., Even P., Lafont R., Dioh W., Veillet S., Tomé D., Huneau J.F., Hermier D., Quignard-Boulangé A. (2014). Quinoa Extract Enriched in 20-Hydroxyecdysone Affects Energy Homeostasis and Intestinal Fat Absorption in Mice Fed a High-Fat Diet. Physiol. Behav..

[59] Li L., Lietz G., Seal C.J. (2024). Effects of Quinoa Intake on Markers of Cardiovascular Risk: A Systematic Literature Review and Meta-Analysis. Food Rev. Int..

[60] Atefi M., Mirzamohammadi S., Darand M., Tarrahi M.J. (2022). Meta-Analysis of the Effects of Quinoa (*Chenopodium quinoa*) Interventions on Blood Lipids. J. Herb. Med..

[61] Sharifi-Zahabi E., Nasiri N., Hajizadeh-Sharafabad F., Mohammadi S., Shahbazi S., Salehi N. (2025). High-Density Lipoprotein Particles Are Associated with the Risk of Mortality from All-Causes and Cardiovascular Diseases (CVDs) in Patients with CVD: A Systematic Review and Meta-Analysis Study. Lipids Health Dis..

[62] Karimian J., Abedi S., Shirinbakhshmasoleh M., Moodi F., Moodi V., Ghavami A. (2021). The Effects of Quinoa Seed Supplementation on Cardiovascular Risk Factors: A Systematic Review and Meta-Analysis of Controlled Clinical Trials. Phytother. Res..

[63] Begum K., Khan I., Al-Rizeiqi M.H., Johnson S.K., Madi Almajwal A. (2025). Effect of Buckwheat-Containing Bread on Postprandial Glycemia, Appetite, Palatability, and Gastrointestinal Well-Being. Food Sci. Nutr..

[64] Cesur F., Hatunoglu H.N., Saglam G. (2025). Can There Be Differences in Blood Glucose Fluctuations with Consumption of Cornbread in Obesity and Normal-Weight Individuals: A Randomized Controlled Trial. Plant Foods Hum. Nutr..

[65] Ray A., Das M. (2025). Modulatory Implications of Quinoa in Therapeutic Management of Obesity and Metabolic Syndrome. Food Biosci..

[66] Lee M.S., Shin Y., Jung S., Kim S.Y., Jo Y.H., Kim C.T., Yun M.K., Lee S.J., Sohn J., Yu H.J. (2017). The Inhibitory Effect of Tartary Buckwheat Extracts on Adipogenesis and Inflammatory Response. Molecules.

[67] Giménez-Bastida J.A., Zieliński H. (2015). Buckwheat as a Functional Food and Its Effects on Health. J. Agric. Food Chem..

[68] Sanders L.M., Zhu Y., Wilcox M.L., Koecher K., Maki K.C. (2023). Whole Grain Intake, Compared to Refined Grain, Improves Postprandial Glycemia and Insulinemia: A Systematic Review and Meta-Analysis of Randomized Controlled Trials. Crit. Rev. Food Sci. Nutr..

[69] Reynolds A.N., Akerman A.P., Mann J. (2020). Dietary Fibre and Whole Grains in Diabetes Management: Systematic Review and Meta-Analyses. PLoS Med..

[70] Schulze M.B., Stefan N. (2024). Metabolically Healthy Obesity: From Epidemiology and Mechanisms to Clinical Implications. Nat. Rev. Endocrinol..

