# Supplementary material for: Comparative Effects of Quinoa, Buckwheat, Bulgur, and Rice on Cardiometabolic Health in Adults with Overweight or Obesity: A Randomized Controlled Study
Source: Metabolites. 2026 Aug 29;16(9):628. doi: 10.3390/metabo16090628 (PMC13609460; doi:10.3390/metabo16090628)
Supplement: Supplementary file 1 [file metabolites-16-00628-s001.zip › metabolites-4503184-supplementary.pdf]

The CONSORT reporting checklist

For checking that reports of randomised trials can be understood and used by everyone

	Item Description	Location (or reason for not reporting)
**Title and Abstract**		
1a. Title	Identification as a randomised trial.	Page 1, Title
1b. Structured Abstract	Structured summary of the trial design, methods, results, and conclusions.	Page 1, Abstract
**Open Science**		
2. Trial Registration	Name of trial registry, identifying number (with URL) and date of registration.	Title page ClinicalTrial registration, Methods; Paragraph 1
Protocol and statistical analysis plan	Where the trial protocol and statistical analysis plan can be accessed.	Title page ClinicalTrial registration, Methods; Paragraph 1
4. Data sharing	Where and how the individual de-identified participant data (including data dictionary), statistical code and any other materials can be accessed.	Title Page Clinical trial registration, Data Availability
**5. Funding and Conflicts of Interest**		
5a. Funding	Sources of funding and other support (eg, supply of drugs), and role of funders in the design, conduct, analysis, and reporting of the trial.	Title Page; Funding<br>Main text; Funding
5b. Conflicts of interest	Financial and other conflicts of interest of the manuscript authors.	Title page; Declaration of Interest Statement<br>Main text; Declaration of Interest Statement
**Introduction**		
6. Background and rationale	Scientific background and rationale.	Introduction
7. Objectives	Specific objectives related to benefits and harms.	Introduction
**Methods**		

8. Patient and public involvement	Details of patient or public involvement in the design, conduct and reporting of the trial.	Methods, Participants
9. Trial Design	Description of trial design including type of trial (eg, parallel group, crossover), allocation ratio, and framework (eg, superiority, equivalence, non-inferiority, exploratory).	Methods, Randomization
10. Changes to trial protocol	Important changes to the trial after it commenced including any outcomes or analyses that were not pre-specified, with reason.	There are no changes in the trial after commencement.
11. Trial Setting	Settings (eg, community, hospital) and locations (eg, countries, sites) where the trial was conducted.	Methods, Participants
12. Eligibility Criteria		
12a. Participants	Eligibility criteria for participants.	Methods, Participants; paragraph 1
12b. Other	If applicable, eligibility criteria for sites and for individuals delivering the interventions (eg, surgeons, physiotherapists).	Methods, Participants; paragraph 1
13. Intervention and comparator	Intervention and comparator with sufficient details to allow replication. If relevant, where additional materials describing the intervention and comparator (eg, intervention manual) can be accessed.	Methods, Preparation and Instructions for the Consumption of Pseudocereals and Cereals, paragraph 1
14. Outcomes	Prespecified primary and secondary outcomes, including the specific measurement variable (eg, systolic blood pressure), analysis metric (eg, change from baseline, final value, time to event), method of aggregation (eg, median, proportion), and time point for each outcome.	Methods, paragraph 1 (primary and secondary outcomes)<br>Methods, Anthropometric Measurements, Blood Pressure and Biochemical Assessment (measurement variables)<br><br>Methods; Statistical Analysis (analytic metrics and method of aggregation)
15. Harms	How harms were defined and assessed (eg, systematically, non-systematically).	Methods; Dietary Intake<br>Results; Adverse effects
16. Sample Size		
16a. How sample size was determined	How sample size was determined, including all assumptions supporting the sample size calculation.	Methods, Sample Size
16b. Interim analyses and stopping criteria	Explanation of any interim analyses and stopping guidelines.	There are no interim analysis and no stopping guidelines because the intervention was not related to expected harm to participants.

17. Randomisation		
17a. Sequence Generation	Who generated the random allocation sequence and the method used.	Methods, Randomization
17b. Type of Randomisation	Type of randomisation and details of any restriction (eg, stratification, blocking, and block size).	Methods, Randomization
18. Allocation concealment mechanism	Mechanism used to implement the random allocation sequence (eg, central computer/telephone; sequentially numbered, opaque, sealed containers), describing any steps to conceal the sequence until interventions were assigned.	Methods, Randomization
19. Implementation	Whether the personnel who enrolled and those who assigned participants to the interventions had access to the random allocation sequence.	Methods, Randomization
20. Blinding		
20a. Who was blinded	Who was blinded after assignment to interventions (eg, participants, care providers, outcome assessors, data analysts).	There is no blinding due to nature of dietary interventional studies.
20b. How blinding was achieved	If blinded, how blinding was achieved and description of the similarity of interventions.	There is no blinding due to nature of dietary interventional studies.
21. Statistical methods		
21a. Comparing groups	Statistical methods used to compare groups for primary and secondary outcomes, including harms.	Methods, Statistical Analysis;
21b. Definition of who is included in each analysis	Definition of who is included in each analysis (e.g., all randomized participants), and in which group.	Results, paragraph 1
21c. Missing Data	How missing data were handled in the analysis.	Results, paragraph 1
21d. Additional Analyses	Methods for any additional analyses (eg, subgroup and sensitivity analyses), distinguishing pre-specified from post hoc.	Methods, Statistical Analysis
**22. Participant flow, including flow diagram**		
22a. Participant Numbers	For each group, the numbers of participants who were randomly assigned received intended intervention, and were analysed for the primary outcome.	Methods, Figure 1 Flow Chart Results
22b. Losses and exclusions	For each group, losses and exclusions after randomisation, together with reasons.	Methods, Figure 1 Flow Chart Results, paragraph 1
**23. Recruitment**		

23a. Dates	Dates defining the periods of recruitment and follow-up for outcomes of benefits and harms.	Methods, Participants
23b. Reasons for stopping recruitment	If relevant, why the trial ended or was stopped.	The trial was not ended or stopped.
**24. Intervention and comparator delivery**		
24a. As Administered	Intervention and comparator as they were actually administered (eg, where appropriate, who delivered the intervention/comparator, whether participants adhered, whether they were delivered as intended (fidelity)).	Methods; Preparation and Instructions for the Consumption of Pseudocereals and Cereals
24b. Concomitant Care	Concomitant care received during the trial for each group.	Methods, Participants
25. Baseline Data	A table showing baseline demographic and clinical characteristics for each group.	Results; Table 2
26. Numbers analysed, outcomes, and estimation	For each primary and secondary outcome, by group:<br>• the number of participants included in the analysis.<br>• the number of participants with available data at the outcome time point.<br>• result for each group, and the estimated effect size and its precision (such as 95% confidence interval).	Results, paragraph 1<br>For primary outcomes: Table 3,4 and 5.<br><br>For secondary outcomes: Table 6
	• for binary outcomes, presentation of both absolute and relative effect size.	
27. Harms	All harms or unintended events in each group.	Results; Adverse effects
28. Ancillary Analyses	Any other analyses performed, including subgroup and sensitivity analyses, distinguishing pre-specified from post hoc.	Results, Title 3.3 Anthropometrical Measurements, Title 3.4. Dietary Intakes
**Discussion**		
29. Interpretation	Interpretation consistent with results, balancing benefits and harms, and considering other relevant evidence.	Discussion, paragraphs 2,3,4,5,6,7,8,9
30. Limitations	Trial limitations, addressing sources of potential bias, imprecision, generalisability, and, if relevant, multiplicity of analyses.	Discussion, Paragraph 9
